# Life Cycle Assessment of Solvothermal Zeolitic Imidazolate Framework‐8 Synthesis: Is the Substitution of *N*,*N*‐Dimethylformamide with Glycerol Carbonate Environmentally Sustainable?

**DOI:** 10.1002/cssc.202502019

**Published:** 2025-11-16

**Authors:** Alessandra Sessa, Eleonora Rossi, Prisco Prete, Fabrizio Passarini, Masaki Itatani, Federico Rossi, Istvan Lagzi, Pierandrea Lo Nostro, Daniele Cespi, Raffaele Cucciniello

**Affiliations:** ^1^ Department of Chemistry and Biology “Adolfo Zambelli” Salerno University Fisciano Italy; ^2^ Department of Industrial Chemistry “Toso Montanari” Alma Mater Studiorum ‐ University of Bologna Bologna Italy; ^3^ Department of Chemistry Faculty of Science Hokkaido University Sapporo Japan; ^4^ Department of Physical Sciences, Earth and Environment University of Siena Siena Italy; ^5^ Department of Physics Institute of Physics Budapest University of Technology and Economics Budapest Hungary; ^6^ HUN‐REN–BME Condensed Matter Physics Research Group Budapest University of Technology and Economics Budapest Hungary; ^7^ Department of Chemistry “Ugo Schiff” University of Florence Firenze Italy

**Keywords:** biobased, glycerol carbonate, life cycle assessment (LCA), solvothermal, zeolitic imidazolate framework (ZIF‐8)

## Abstract

In this work, a comparative life cycle assessment of the solvothermal synthesis of zeolitic imidazolate framework (ZIF‐8) is reported. ZIF‐8 is listed among the most representative structures of ZIFs, an emerging subclass of metal–organic frameworks (MOFs). ZIF‐8 is commonly synthesized through the solvothermal method, and the election solvents are currently *N*, *N*‐dimethylformamide (DMF) and water, both allowing the production of highly crystalline ZIF‐8 with a high specific surface area. Considering the recently introduced severe restrictions on the use of DMF as defined very recently by European Chemical Agency, an innovative synthetic procedure in the biobased and nontoxic glycerol carbonate (GlyC) was reported. This approach delivers mesoporous ZIF‐8 with high crystallinity and high specific surface area. The present study suggests a cradle‐to‐gate life cycle assessment of the laboratory scale ZIF‐8 synthesis, comparing the same process in DMF and GlyC in terms of their environmental impacts. The results help to demonstrate that the respect of the 7^th^ principle of green chemistry (using GlyC instead of DMF) does not necessarily embody, at present, a sustainable choice.

## Graphical abstract

Green Chemistry For Sustainability: The life cycle assessment approach reveals that replacing a toxic solvent (e.g., *N*, *N*‐dimethylformamide) with a bio‐derived, safer alternative (e.g., glycerol carbonate), in accordance with the 7^th^ principle of green chemistry, is not sufficient alone to ensure environmental sustainability.

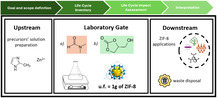

**Abbreviations**
avp: avoided products
CAGR: Compound Average Growth Rate
CED: Cumulative Energy Demand
DMC: Dimethyl Carbonate
DMF: *N,N* ‐ Dimethylformamide
GlyC: Glycerol Carbonate
LCA: Life Cycle Assessment
LCIA: Life Cycle Impact Assessment
MeOH: Methanol
MOF: Metal‐Organic Framework
SSbD: Safe and Sustainable by Design
Z‐DMF: ZIF‐8 synthesis in DMF
Z‐GlyC‐avp: ZIF‐8 synthesis in GlyC‐avp
Z‐GlyC‐base: ZIF‐8 synthesis in GlyC‐base
Z‐GlyC‐best: ZIF‐8 synthesis in GlyC‐best
Z‐GlyC‐wco: ZIF‐8 synthesis in GlyC‐wco
ZIF: Zeolitic Imidazolate Framework
wco: Waste Cooking Oil

## 1 Introduction

Over the last few decades, the scientific community has increasingly focused on researching, studying, and developing innovative technologies to support the transition toward a zero‐carbon footprint and environmental sustainability. Concepts such as circularity, climate neutrality, safer chemicals, economic growth, and technological innovation, as well as an increasing social awareness, are the cornerstones requested by the safe and sustainable by design (SSbD) framework promoted by the European Commission [1, 2–3].

The SSbD strategy strives to (a) pursue the innovation of processes toward a sustainable industrial transition, (b) limit the usage of harmful substances, (c) systematically remove them from material and product cycles, and (d) reduce the effects on health, climate, and environment throughout the whole product life cycle [3]. Furthermore, all the current scientific efforts are made to guarantee a benign by design (B‐B‐D) society, with the aim of reducing inequalities and social conflicts [4, 5].

From this perspective, the life cycle assessment (LCA) methodology is a handy tool that allows for contextualizing specific chemical or technological data within the broader perspective of the entire product life cycle, from the extraction of raw materials to its final disposal and waste management at the end of life [6, 7–8].

LCA relies on a life cycle thinking (LCT) perspective aimed at providing support in integrating sustainability into policy making under a holistic point of view [9].

Particularly, LCA applied to early‐stage [10] processes results as a relevant indicator for the technology development, even if at a still preliminary stage.

Generally, this approach has been used at the decision‐making stage to verify if green chemistry principles apply to the case study [11]. For instance, a recent study has already used a simplified assessment at an early stage on a pilot scale to evaluate two biobased routes for the production of maleic anhydride, starting from furfural and n‐butanol, respectively, to assess their corresponding benefits and drawbacks before the potential industrial scale‐up [6].

In addition, this work aims to align with the principles of green chemistry, particularly the 7^th^ principle [12], which strongly encourages the use of renewable resources instead of chemicals derived from fossil resources.

LCA serves as a reliable support in the decision‐making stage, since it allows for identifying potential hotspots of the process, to address the research toward targeted improvements of the process itself before it is implemented on a large scale [13].

Assessing where improvements can be made when a process is still at the laboratory stage can be a key to understanding the environmental improvement potential, forming the basis of ecodesign [14]. In fact, identifying and addressing hotspots is crucial for maximizing the potential environmental benefits [15].

In summary, evaluating innovative synthetic processes in terms of their actual environmental impact, followed by a critical comparison with the existing methods through international standards, has become an established practice to verify the effectiveness of the new proposed process.

To date, in our opinion, a recurring misunderstanding persists in the current literature, where the term *green* is often equated with *sustainable* without any actual evaluation of the environmental sustainability. This misconception is commonly found, particularly in everyday life, as consumers tend to trust the green claims displayed on products, which are frequently not authentic but rather misleading, causing consumers to become victims of the widespread phenomenon known as *greenwashing*. EU is actually fighting against this issue through a proposed new law about green claims, in order to ensure greater transparency and credibility from the companies, making the users more aware of their purchases [16]. Moreover, adherence to green chemistry principles is frequently presented as a pathway to sustainability, despite the absence of a transparent methodology capable of giving concrete meaning to these claims. As initially defined by Paul Anastas and recently revitalized through the “Green Chemistry for Sustainability” program [12], green chemistry aims to enhance sustainability. However, following one or more green chemistry principles does not automatically ensure that a particular choice is sustainable at present.

In summary, replacing a molecule derived from fossil sources with the same molecule obtained from biomass does not constitute a sustainable substitution unless life cycle tools are applied to provide a proper evaluation. In fact, it is often prematurely assumed that the bio‐based alternative is inherently sustainable, with attention placed solely on the origin of the molecule. This perspective overlooks critical aspects such as the quantity of raw materials or energy required for a specific process, as well as the amount and nature of solvents used. The outcome clearly depends also on the maturity of the specific process or technology.

Furthermore, although a series of green solvents have emerged, the environmental evaluation of solvent production remains in its early stage, and consequently, their real sustainability needs to be assessed [17].

With the ambition to contribute to this research field, in this work, a LCA approach has been adopted to assess the whole life cycle of a laboratory‐scale synthesis of zeolitic imidazolate framework (ZIF‐8) in glycerol carbonate (GlyC) through the solvothermal method [18]. GlyC has attracted growing attention in recent years thanks to its chemical and physical properties, which make this highly reactive molecule a promising green starting material for organic synthesis [19, 20]. Recently, GlyC has been innovatively reported in electrolyte formulations for supercapacitors and potassium‐ion batteries [21].

All the steps from the precursors’ preparation up to final disposal of byproducts were considered, as well as all the energy consumption involved in each stage. The proposed ZIF‐8 preparation in GlyC was also compared, in terms of environmental performance, with an already existing procedure based on a different solvent, *N*,*N*‐dimethylformamide (DMF), which is the preferred route compared to the state‐of‐the‐art [22]. All the above‐mentioned preparation protocols involve using zinc acetate (or nitrate) and 2‐methylimidazole as ZIF‐8 precursors.

## 2 Background: Industrial Relevance and Synthesis of ZIF‐8

ZIF‐8 is one of the most representative materials for the broader class of ZIFs, a subclass of metal–organic frameworks (MOFs). ZIFs are particularly attractive because they exhibit the benefits of both MOFs and zeolites, providing excellent crystallinity, high porosity, and impressive chemical and thermal stability [23, 24]. Thanks to their properties and high surface area, ZIFs are widely used in catalysis and for separating gaseous mixtures, particularly for hydrogen and methane/carbon dioxide separations [25, 26–27]. They also play a significant role in chemiluminescence due to their tunable adsorption capabilities and are employed in controlled drug release systems due to their sensitivity to pH changes [28].

Thanks to these outstanding properties, the ZIF market was valued at USD 1.2 billion in 2024 and is projected to reach USD 3.5 billion by 2033, growing at a compound annual growth rate (CAGR) of 15.5% from 2026 to 2033. Within this market, ZIF‐8 stands out as the leading material, accounting for over 35% of the total market share [29].

Traditionally, ZIF‐8 is synthesized through the solvothermal method, which involves the reaction between a zinc salt (e.g., Zn(CH_3_COO)_2_, Zn(NO_3_)_2_, ZnCl_2_), and 2‐methylimidazole, according to the Reaction (1) :

(1) $${\mathrm{ZnX}}_{2}+2(2-\mathrm{MIm})\;\to \;\mathrm{ZIF}-8+2\mathrm{HX}$$

Some studies have compared the outcomes achieved with different solvents and synthetic methods to evaluate how each solvent influenced the resulting ZIF‐8, leading to variations in morphology, particle size, crystallinity, and surface area, all of which are crucial for specific applications [30].

Tezerjani et al. reported the synthesis of ZIF‐8 in DMF at 140 °C for 24 h, achieving a yield of 30.5% of a high‐crystalline ZIF‐8 [22]. To date, the solvothermal synthesis in DMF is the most consolidated approach in the presence of an organic solvent.

However, in 2020, the European Chemicals Agency (ECHA) published its guidelines concerning the use of DMF, classifying it as a hazardous substance, and introduced more severe restrictions, especially concerning occupational exposure and use in consumer products [31]. Efforts should be made to reduce the use of DMF in the early‐stage process to avoid potential regulatory issues. In this context, the substitution of DMF where possible is strongly encouraged, and this choice must be closely accompanied and supported by the design and development of new methodologies, so that the process can be optimized with new solvents to replace DMF [32].

In this scenario, Cravillon et al. showed how to prepare ZIF‐8 crystals at room temperature by replacing DMF with MeOH, achieving a yield of 23% [33].

Notwithstanding the relevance of ZIF‐8 as an advanced material and the extensive literature concerning its synthesis, only a few papers have discussed its preparation from a LCA perspective.

An interesting work presenting an LCA on the synthesis of 1 kg of ZIF‐8 for CO_2_ capture compared various synthesis methods (microwave, sonochemical, mechanochemical, dry‐gel conversion, and aqueous system) and revealed that the mechanochemical approach is the least impactful. Furthermore, since for most methods the solvent needed for washing the obtained ZIF‐8 is DMF in large amount, and it has the largest contribution for most of the 18 impact categories (ReCiPe, midpoint) in the life cycle impact assessment phase (LCIA). Regarding the solvothermal method, replacing the solvent of DMF with methanol remarkably reduced the influence on human health, ecosystem, and resources, and the authors recommend replacing traditionally used solvents (i.e., DMF) with greener and biobased alternatives [34].

We recently contributed to this request by testing GlyC as an alternative solvent to DMF to carry out the synthesis of ZIF‐8 [35]. To the best of our knowledge, this is the first example of ZIF‐8 preparation in a nontoxic, biobased, and biodegradable organic solvent. GlyC owns a high dielectric constant and dipole moment like DMF, but also possesses biodegradability and low toxicity [36, 37]. GlyC was found to be a promising solvent for this reaction, and at [Zn(OAc)_2_]/[Hmim] = 10:20 ratio, *T* = 100°C, *t* = 24 h, [NaOH] = 10 mM, the mass yield of the product was 57.1%. Under these conditions, ZIF‐8 was synthesized with excellent crystallinity and a characteristic mesoporous isotherm (*S*_BET_ = 660 m^2^ g^−1^; *V*_pore_ = 0.58 cm^3^ g^−1^) [35].

## 3 Experimental Part

Synthesis and characterization of GlyC are reported in the S1 section of the Supporting Information.

### 3.1 Environmental Assessment: Functional Unit, Goal, Scope, and Boundaries

LCA is a widely validated methodology governed by ISO 14 040 [38]‐14 044 [39] standards [40] for assessing the potential environmental impacts of a product or process throughout its entire life cycle, from raw material extraction up to manufacturing and final disposal.

The aim of the study is to provide a cradle‐to‐gate LCA referring to the synthesis of 1 g of ZIF‐8 (FU), by comparing the same reaction carried out in DMF and in GlyC, respectively. The comparative approach is helpful to grasp the environmental performances of the proposed method in relation to the most efficient solvothermal synthesis reported in the literature, in terms of yield and quality of the desired product. All collected data in input and output fluxes (both materials and energy) were normalized to the functional unit, and linear scaling was applied (moving from micrograms to grams) [41].

Mass balance for each scenario is shown in Section S2 of the Supporting Information As previously mentioned, the proposed study refers to reactions conducted on a laboratory scale and for this reason, the present study can be classified as an early stage‐LCA.

In this sense, the LCA tool can be valuable in identifying environmental hotspots at early technological development, guiding developers to focus their efforts on these critical areas [10].

Consequently, the maturity of the process can be expressed by defining the process as having a technology readiness level (TRL) of 4, meaning that the process is reproducible and thus validated at the laboratory scale [42].

### 3.2 Scenario Description and Further Assumptions

For each scenario, the system boundaries cover the production chain from the raw material employed to the final product at laboratory scale (=laboratory gate). It is also important to note that the disposal of ZIF‐8 after its use was not considered within the scope of this work. Nevertheless, ZIF‐8 are commonly known as a stable material under ambient conditions and after incineration, it forms ZnO as a by‐product, which could be reused for other applications in addition to other reaction products [43].

The DMF‐based synthesis (Z‐DMF) is depicted in detail and displayed in the flowchart in Figure S2.1 of the Supporting Information.

**FIGURE 1:**
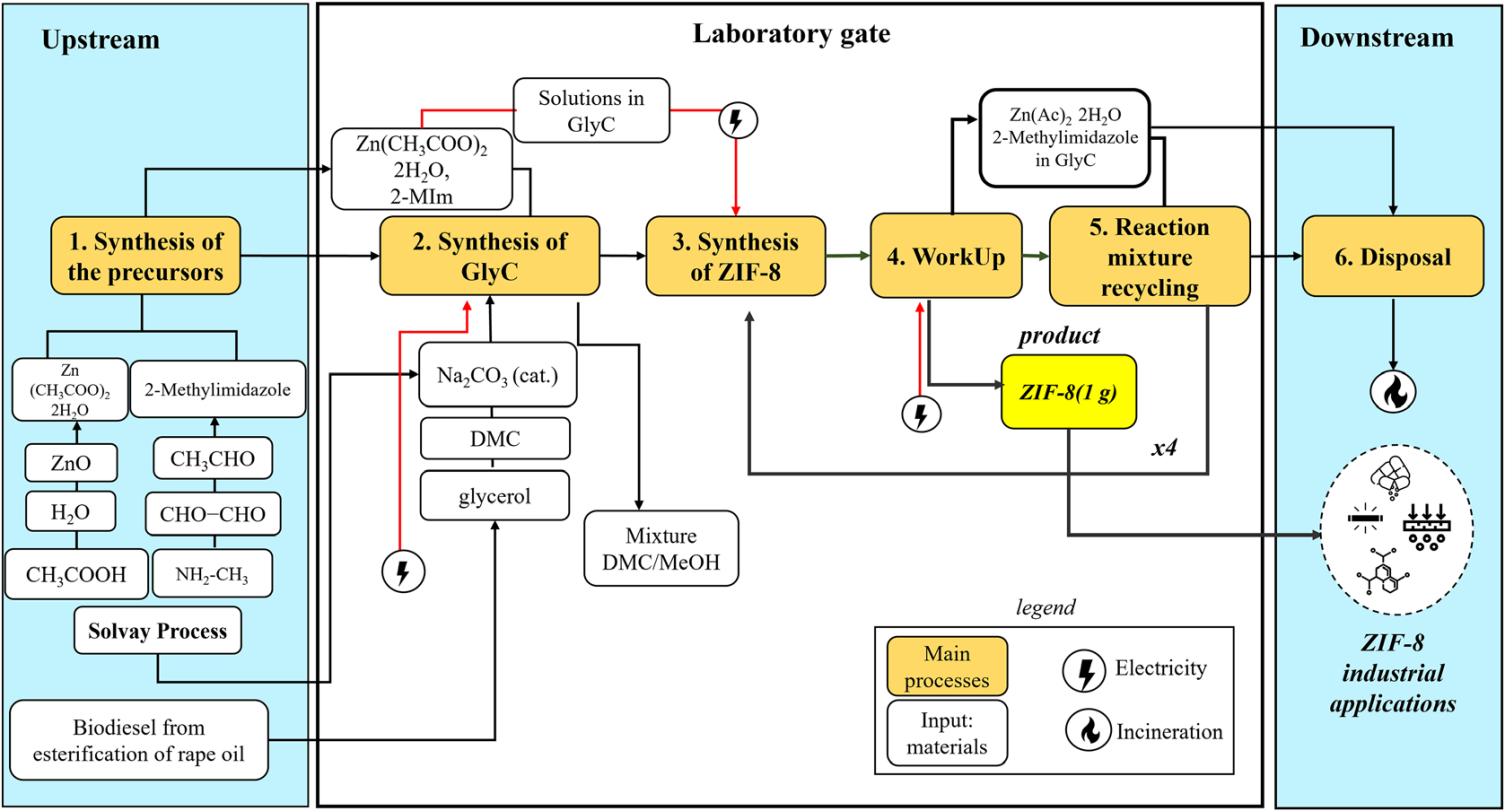
Flowchart and system boundaries for the Z‐GlyC‐base scenario.

To design the GlyC baseline scenario (Z‐GlyC‐base), all the considered mass inputs and energy fluxes were calculated by considering both the main reaction with the consecutive four recycling tests for a total of five reactions, considering the yield of each synthesis, and the FU was scaled on 1 g of ZIF‐8. The flowchart covering the whole cradle to gate process is shown in detail in Figure 1. Biodiesel production process was selected as glycerol's main source, through esterification of rapeseed oil (*Glycerin {Europe without Switzerland} | esterification of rape oil | APOS*, *U*)*.* Indeed, at the moment, biodiesel production from rapeseed oil accounts for 80% of the European biofuel market [44]. In all the scenarios created, GlyC is assumed to be obtained through transcarbonation of glycerol with DMC [45].

Subsequently, starting from Z‐GlyC‐base, additional scenarios were hypothesized based on modifications regarding both the glycerol source and the final treatment of the generated by‐product (DMC/MeOH mixture).

In this context, it is relevant to highlight that the valorization of waste cooking oil (WCO) for producing glycerol can be classified as a virtuous example of circular economy, as it is currently one of the largest industrial wastes [44].

In fact, WCO can be considered as an emerging feedstock for biodiesel production as it has no geographical constraint, also, the used cooking oil market was valued at USD 6.5 billion in 2020 globally and is expected to grow at a compounded average growth rate (CAGR) of 5.1% during 2021–2028 [46].

The first improved scenario was called “GlyC‐wco,” assuming WCO as an alternative source to be explored for reducing the impacts related to GlyC synthesis [46].

Another improvement to the baseline scenario was linked to the disposal of the byproducts deriving from the transcarbonation route for the GlyC synthesis.

The 1:1 in mol mixture of DMC and MeOH is recovered after the distillation, and in the Z‐GlyC‐base scenario, it is sent to incineration.

A potential reuse of this mixture could be an example of waste valorization, where both methanol and DMC appear as avoided products, and it could also influence the impacts in a positive sense. This redesigned scenario is depicted as “GlyC‐avp”.

These two new optimized scenarios were initially evaluated individually to quantify the influence of each parameter on the overall impacts for GlyC synthesis, and subsequently they were condensed into one extra scenario, called “GlyC‐best”.

**FIGURE 2:**
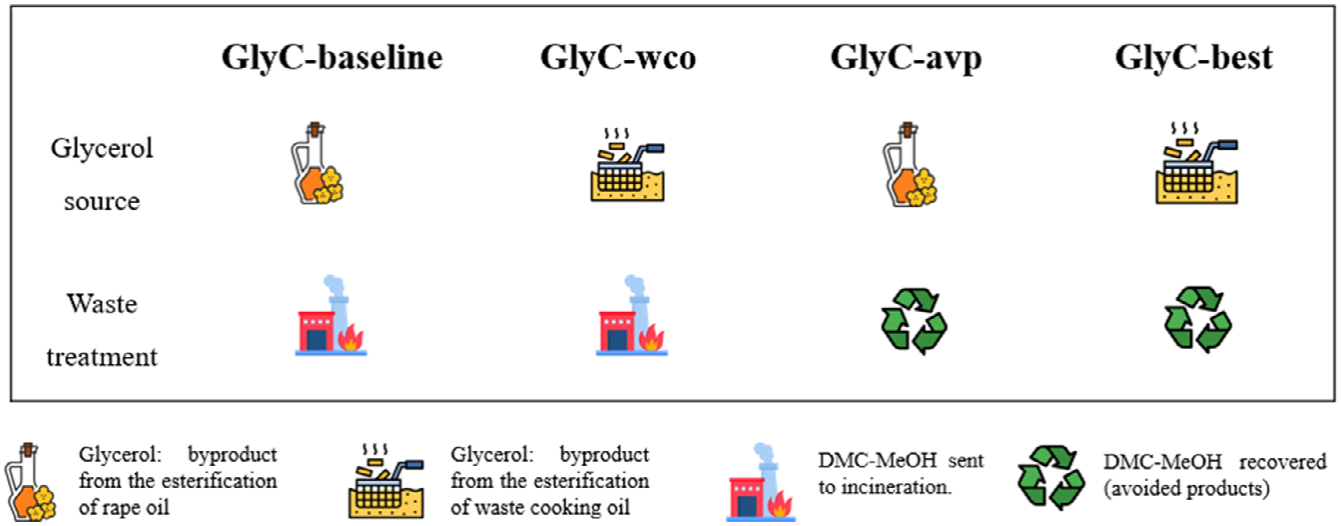
Description of all the GlyC‐based scenarios involved in the analysis.

To simplify the distinction between the baseline scenario and the new scenario just hypothesized, all of them are described in detail and schematized in the following Figure 2.

### 3.3 Data Acquisition and Life Cycle Inventory

ZIF‐8 was prepared in GlyC as a solvent and fully characterized as described in our previous work [35].

The recyclability of the solvent was tested and found to be effective for five consecutive cycles, as previously demonstrated [35].

As a result, the inventory compilation phase involves both primary and secondary data. The former were considered in the case of direct measurements, e.g., for the proposed Scenario, Z‐GlyC‐base, while for Z‐DMF, secondary data were extrapolated from literature [22].

For each process, the most accurate and representative dataset available in the *ecoinvent* database [47] was selected. In cases where no suitable dataset existed, custom models were developed based on stoichiometric calculations and literature or patent sources. Specifically, the production of 2‐methylimidazole was adapted from the existing imidazole dataset by substituting formaldehyde with acetaldehyde and ammonia with methylamine, in line with an alternative synthesis route. Zinc nitrate was modeled according to the reaction between zinc oxide and nitric acid, while zinc acetate was modeled based on the synthesis pathway described in a patent [48]. As a result, the Z‐GlyC‐base scenario is characterized by a relatively low uncertainty degree. Anyway, some assumptions were made, especially for the punctual energy consumption calculation.

First of all, since all the reactions described refer to standard laboratory equipment, the energy consumption is based exclusively on electricity use and, for example, no heat recovery or direct use of industrial steam is considered.

For calculating energy consumption, the *“Electricity*, *low voltage {GLO}| market group for | APOS*, *U”* power of a standard laboratory heating plate was considered. In this context, the electricity consumption (kWh) was calculated by multiplying the power of the instrument (kW) by the time of use (h) [49]. The power of the instruments was calculated considering the actual operating temperature. In fact, when an instrument was used at a temperature lower than the maximum, a corrective factor Δ*T*_working_/Δ*T*_max_ was introduced [13].

The comprehensive energy calculation within the appropriate instrument power allocation is well described in the data sheet in Section S4 of the supporting information.

The same instruments (heating plate, vacuum pump, centrifuge, ultrasonic cleaner machine, and oven) used directly in scenario Z‐GlyC‐base were associated with Z‐DMF scenario, which is based on data extracted from the literature, respectively [22]. Furthermore, the same method for calculating the actual energy consumption was applied to all three scenarios. In this regard, it is important to specify that, while the conditions of scenario Z‐GlyC‐base are well known, higher levels of uncertainty are associated with the Z‐DMF scenario.

### 3.4 Database and Software

The ecoinvent [47] database (*v.3.7.1*) was used for the background data, and “market for” scenarios were selected to account for the impacts related to average transportation distances. In fact, the market dataset represents all activities related to a product within a specific region, including average transport and additional inputs to compensate for trade and transport losses. The following selection criteria were applied: the geographic context is international, so whenever possible, global {GLO} provider was prioritized, or European {RER} provider when {GLO} was unavailable.

The Allocation at Point of Substitution (APOS, U) model was chosen as it is considered the most conservative approach; in fact, it allocates direct impacts based on physical flows, as opposed to other methods such as consequential or cut‐off approaches.

The SimaPro [50] software (*v. 9.2.0.2*) was used as the primary tool for performing the analysis. The harmonized LCIA method ReCiPe 2016 [51] was selected to evaluate potential environmental impacts. This method assesses environmental effects in 18 categories at the midpoint level, or “problem‐oriented” level. The categories include: global warming (GWP); stratospheric ozone depletion (SOD); ionizing radiation (IR); ozone formation, human health (OF_HH); fine particulate matter formation (FPMF); ozone formation, terrestrial ecosystems (OF_TE); terrestrial acidification (TA); freshwater eutrophication (FE); marine eutrophication (ME); terrestrial ecotoxicity (TET); freshwater ecotoxicity (FET); marine ecotoxicity (MET); human carcinogenic toxicity (HCT); human noncarcinogenic toxicity (HNCT); land use (LU); mineral resource scarcity (MRS); fossil resource scarcity (FRS); water consumption (WC). These categories can be further grouped into three *endpoint‐damage* receptors: human health, ecosystem, and resource depletion. The endpoint method enables comparison of different processes based on a cumulative single score, here expressed in millipoint (*mPt*). Another useful tool used to assess environmental impacts was the *Cumulative Energy Demand* [52] (CED V1.11) that allows to quantify the resource consumption (energy and raw material), by defining the renewable and nonrenewable ones.

## 4 Results and Discussion

### 4.1 Life Cycle Impact Assessment

In the first instance, the three well‐described scenarios were compared in terms of their environmental performance across the 18 problem‐oriented impact categories to provide a first immediate and comprehensive assessment of each scenario. In Table 1 results are displayed where a color gradient (ranging from green, through yellow, to red nuances) represents the magnitude of the impacts from minimum (green) to maximum relative value (red) for each midpoint category. Through a quick reading of the table, the following trend can be observed: for the totality of the categories, the proposed Z‐GlyC‐base scenario shows the highest environmental impacts with respect to the Z‐DMF scenario.

**TABLE 1:** Results for the comparison between DMF (Z‐DMF), GlyC baseline(Z‐GlyC‐base), and optimized scenarios (Z‐GlyC‐wco, Z‐GlyC‐avp, and Z‐GlyC‐best) in terms of midpoint impact categories.

Impact category	Z‐DMF	Z‐GlyC‐base	Z‐GlyC‐wco	Z‐GlyC‐avp	Z‐GlyC‐best
GWP	8.29	9.25	8.98	8.35	8.07
SOD	3.53 × 10^−6^	8.16 × 10^−6^	3.42 × 10^−6^	7.91 × 10^−6^	3.17 × 10^−6^
IR	9.76 × 10^−1^	9.37 × 10^−1^	9.28 × 10^−1^	9.06 × 10^−1^	8.97 × 10^−1^
OF_HH	1.78 × 10^−2^	1.88 × 10^−2^	1.79 × 10^−2^	1.77 × 10^−2^	1.68 × 10^−2^
FPMF	1.76 × 10^−2^	1.74 × 10^−2^	1.67 × 10^−2^	1.68 × 10^−2^	1.61 × 10^−2^
OF_TE	1.79 × 10^−2^	1.90 × 10^−2^	1.81 × 10^−2^	1.79 × 10^−2^	1.70 × 10^−2^
TA	2.74 × 10^−2^	3.10 × 10^−2^	2.66 × 10^−2^	2.97 × 10^−2^	2.53 × 10^−2^
FE	4.02 × 10^−3^	4.03 × 10^−3^	3.94 × 10^−3^	3.81 × 10^−3^	3.72 × 10^−3^
ME	4.73 × 10^−4^	2.08 × 10^−3^	3.05 × 10^−4^	2.06 × 10^−3^	2.85 × 10^−4^
TET	5.99	6.19	6.16	5.82	5.80
FET	4.52 × 10^−1^	3.95 × 10^−1^	3.94 × 10^−1^	3.94 × 10^−1^	3.93 × 10^−1^
MET	5.69 × 10^−1^	4.97 × 10^−1^	4.96 × 10^−1^	4.96 × 10^−1^	4.95 × 10^−1^
HCT	4.09 × 10^−1^	3.56 × 10^−1^	3.56 × 10^−1^	3.56 × 10^−1^	3.56 × 10^−1^
HNCT	6.78	5.91	5.90	5.90	5.90
LU	3.47 × 10^−1^	1.38	3.73 × 10^−1^	1.35	3.44 × 10^−1^
MRS	8.75 × 10^−3^	1.27 × 10^−2^	1.17 × 10^−2^	1.11 × 10^−2^	1.01 × 10^−2^
FRS	2.12	2.41	2.37	2.15	2.11
WC	6.63 × 10^−2^	8.26 × 10^−2^	6.78 × 10^−2^	7.79 × 10^−2^	6.31 × 10^−2^

### 4.2 GlyC Baseline Scenario

**FIGURE 3:**
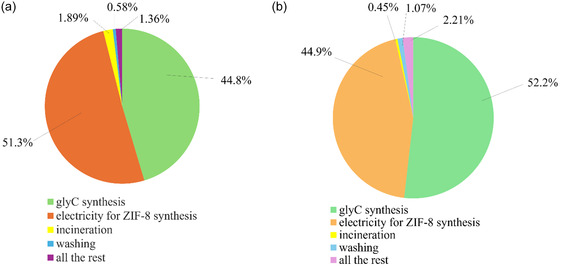
Major contributors to environmental impacts in the case of the Z‐GlyC‐base scenario, in terms of (a) cumulative single score (mPt), and (b) cumulative energy demand (MJ), adopting the ReCiPe 2016 Endpoint and the CED v1.11 approach.

The analysis in terms of a cumulative single score was used to identify the major contributors to environmental impacts in the case of the Z‐GlyC‐base scenario, and the results are shown in Figure 3, expressed in terms of (a) cumulative single score (mPt) and (b) cumulative energy demand (MJ), adopting the ReCiPe 2016 endpoint and the CED approach, respectively. From Figure 3a the predominant contributions are attributed to the synthesis of the solvent (45.4%) and the energy (heating) plate required to carry out the synthesis reaction of ZIF‐8 (48.1%). Regarding the energy contribution due to the ZIF‐8 synthesis reaction, its major impact on the totality of the environmental impacts has also been observed in Z‐DMF, where this contribution accounts for 93.6%, as can be observed in graphs shown in Figure S6.1a of the Supporting Information, where the contribution analysis of Z‐DMF is reported. From the CED analysis (Figure 3b), it comes out that the solvent synthesis becomes an even more predominant aspect, accounting for 52.2% of the total impacts, while the energy consumption for the mixing during the ZIF‐8 synthesis decreases to 44.9%.

**FIGURE 4:**
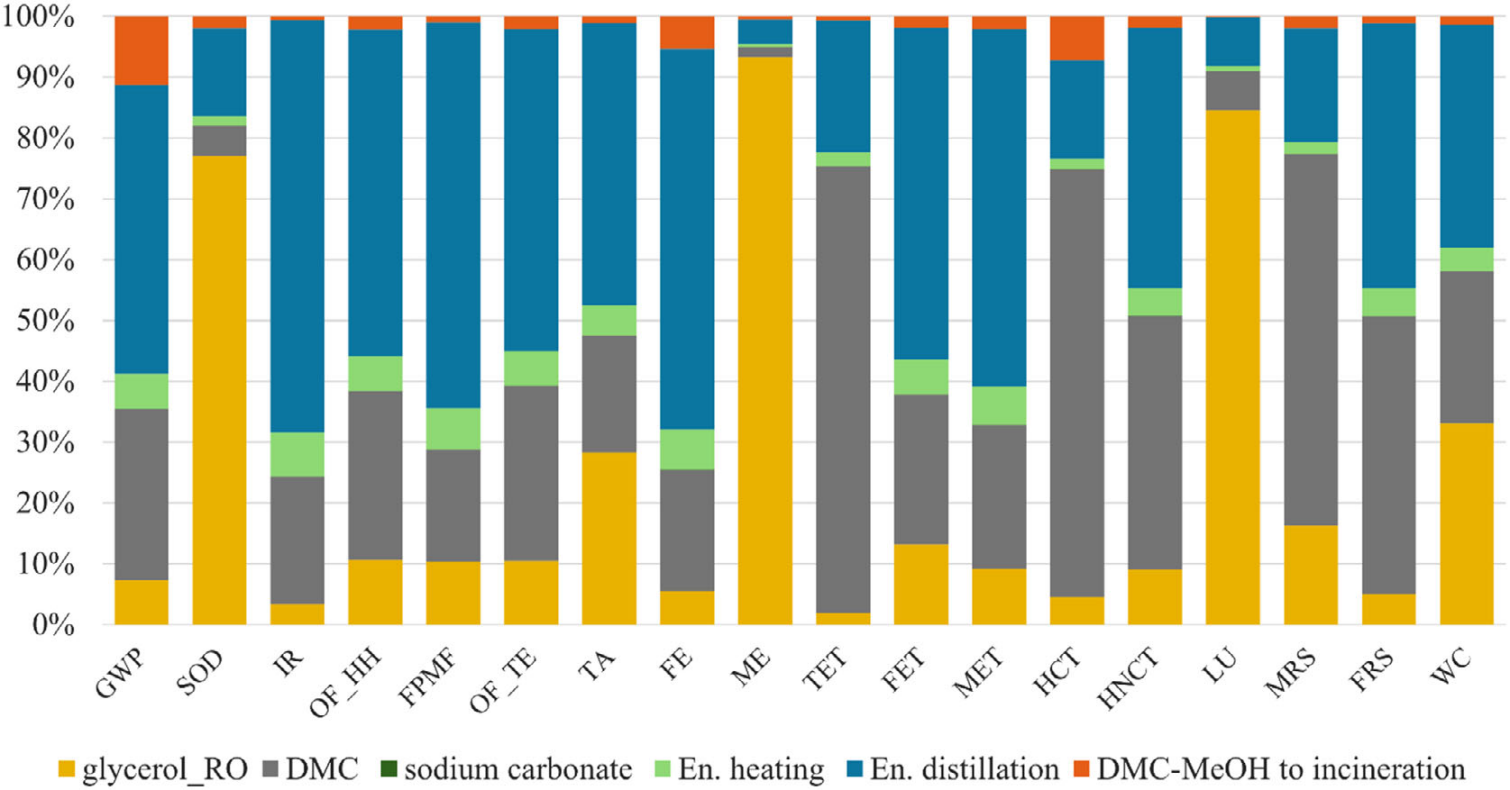
Contribution analysis for GlyC synthesis (ReCiPe midpoint H).

Since the solvent synthesis represents a crucial aspect within the whole process, a contribution analysis for GlyC preparation was therefore conducted and the results are shown in Figure 4.

The energy consumption necessary to perform the GlyC synthesis (especially the one related to the purification step) appears to be the most impactful element (from 36.4% up to 62.3%) for 12 categories over 18, followed by DMC (first contributor, accounting for > 60%, in the cases of TET, HCT, MRS, and glycerol production. The glycerol preparation is the major contributor (> 76%) to SOD, ME, LU.

Solvent purification is generally a crucial aspect that emerges in the life cycle evaluation, as it requires a distillation procedure, a very high energy‐intensive process. In this context, an overestimation of energy consumption is very common since the instrument typically does not operate at a constant maximum power, and in some cases, an allocation by mass or volume should be done [53].

Furthermore, in this context, LCAs based on lab‐scale data present opportunities for optimization, as specific environmental impacts, particularly those related to electricity consumption, may decrease at larger scales due to improved process efficiencies and economies of scale. At larger scales, changes in technology, pricing, and regulatory frameworks may lead to variations in environmental and economic outcomes. These uncertainties make it challenging to produce entirely realistic industrial‐ scale simulations, although their consideration can help improve the accuracy of prospective [54].

### 4.3 Sensitivity Analysis: Optimized Scenarios

Once the efficiency of the process in its current state has been studied from an environmental impact perspective, the new hypothesized scenarios — already depicted and summarized in Table 1 — were studied thoroughly to grasp the environmental effects of the proposed improvements on the global process. A sensitivity analysis was performed using CED and ReCiPe 2016 (endpoint) methods*.*

A shift from the midpoint to the endpoint approach allows to translate specific environmental issues into final damages associated with human health, ecosystem, and resources concerns.

**FIGURE 5:**
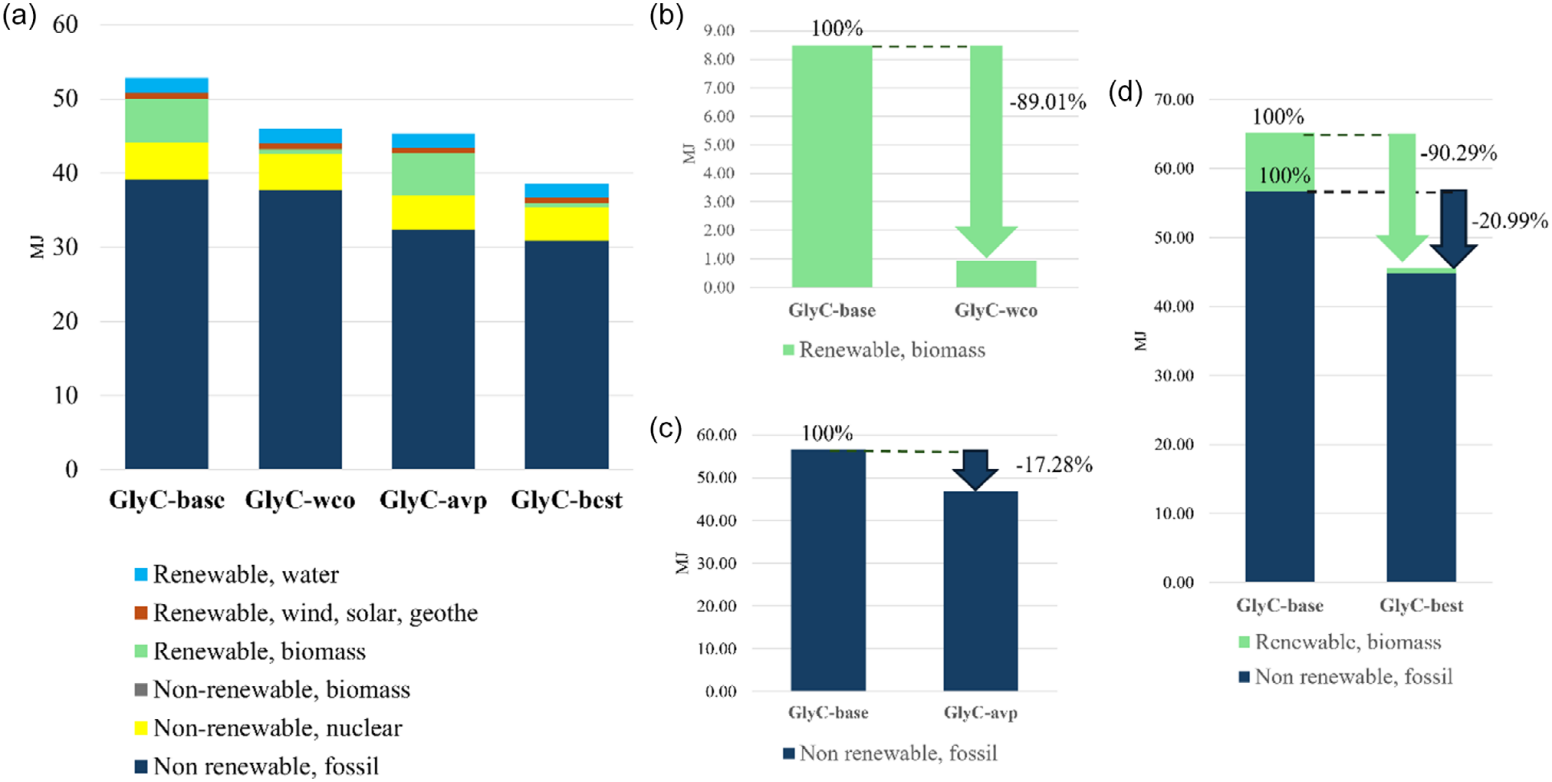
LCIA in terms of single‐issue (Cumulative Energy Demand v1.11) for (a) GlyC‐base, (b) GlyC‐wco, (c) GlyC‐avp, and (d) GlyC‐best.

Comparison of the LCIA insight applying CED method for GlyC‐base (a) and optimized scenarios GlyC‐wco (b), GlyC‐avp (c) and GlyC‐best (d) is shown in Figure 5.

From the CED point of view, glycerol from esterification of GlyC‐wco results to be beneficial for a notable reduction of the renewable fraction of energy deriving from biomass (−94.3%), with respect to the baseline case (GlyC‐base). In the case of MeOH–DMC mixture recovery (GlyC‐avp) instead of its incineration (GlyC‐base), the reduction of the nonrenewable, fossil fraction (−26.3%) is observed.

The best scenario (GlyC‐best) involves a reduction of 68.0% for the nonrenewable‐fossil fraction and 95.6% of the renewable fraction from biomass energy compared with the GlyC‐base scenario. All these reduction percentages are displayed in Figure 5a–d.

**FIGURE 6:**
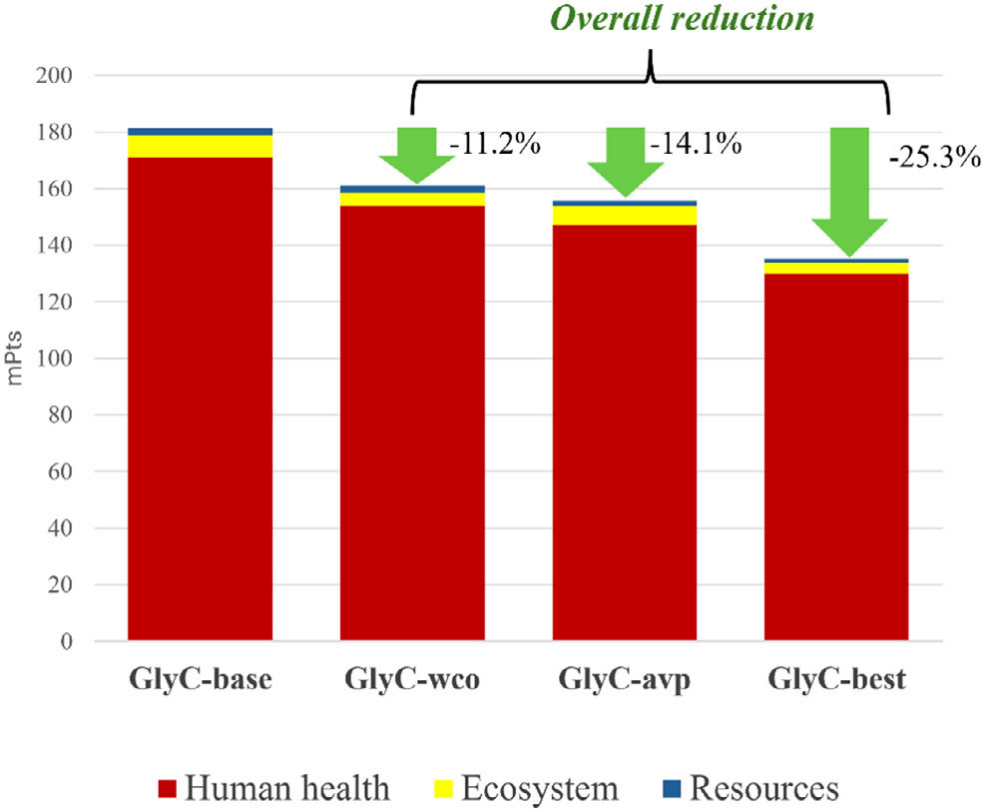
Environmental impacts expressed in terms of single score (mPt) referred to human health, ecosystem, and resources issues according to the ReCiPe 2016 Endpoint (H) V1.08/ World (2010) H method for GlyC‐base, GlyC‐wco, GlyC‐avp, and GlyC‐best.

Additionally, Figure 6 shows the results in terms of a cumulative single score when adopting the ReCiPe 2016 endpoint (H).

The predominant impacts for all the illustrated scenarios are those related to human health damages. As the chart suggests and as it could be reasonably expected, the most beneficial scenario in terms of energy efficiency is GlyC‐best, where both the improvements (glycerol for GlyC synthesis only coming from WCO and DMC and MeOH as avoided products) are applied to the baseline scenario.

The three scenarios (GlyC‐wco, GlyC‐awp, and GlyC‐best), compared to the reference scenario (GlyC‐base), are characterized by a reduction of the total impacts by 11.2%, 14.1%, and 25.3%, respectively.

At this point, a further comparison between the two conventional ZIF‐8 synthesis routes with the one involving GlyC‐best was studied to analyze the effect of the improvements on the overall reaction.

First, all the total scenarios were compared regarding problem‐oriented impacts, as shown in Table 1.

The first improvement—represented by the scenario “Z‐GlyC‐wco”—results in a slight reduction in environmental impacts, a trend similarly observed with the Z‐GlyC‐wco scenario.

A pronounced improvement is discernible exclusively in the comparison between Z‐DMF and Z‐GlyC‐best, with significant reductions observed across 14 out of 18 midpoint impact categories.

The implementation of GlyC‐best throughout the ZIF‐8 synthesis yields more favorable outcomes than the process involving GlyC‐base. These findings support the assertion that, under optimized conditions, the use of GlyC in this synthesis represents a competitive alternative to the conventional application of DMF.

**FIGURE 7:**
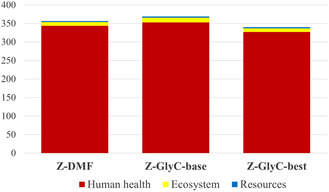
Comparison of ZIF‐8 synthesis of Z‐DMF, Z‐Water, Z‐GlyC‐base, and Z‐GlyC‐best in terms of Single Score [ReCiPe endpoint H V1.08/World (2010) H].

Finally, in Figure 7 the comparison among ZIF‐8 syntheses conducted in DMF, GlyC‐base, and GlyC‐best in terms of single score is reported. Z‐GlyC‐best is characterized by a reduction of 11.9% compared to Z‐GlyC‐base.

Furthermore, since Scenario Z‐GlyC‐best seems to be the most promising compared to the baseline, an uncertainty analysis was carried out to test the robustness of the LCA simulation using the Monte Carlo method.

A data “Pedigree matrix” [55] ‐detailed in Table S2.3.1‐ was employed to determine the uncertainty ranges for the LCI data related to reagents, energy input, and waste streams. The matrix assigns data quality scores on a scale from 1 (best) to 5 (worst), based on various sources of uncertainty that may impact a method's reproducibility and robustness. These sources include data representativeness, acquisition methods, and correlations across time, technology, and geography. Furthermore, the standard deviation (SD) of input and output data for each process was determined using the data quality pedigree matrix, and values are reported in Table S2.3.2 The lognormal statistical distribution, with a 95% confidence interval, was used for the calculations.

Uncertainity analyses performed using the ReCiPe method are reported in the Figures S7.3 and S7.4. The Monte Carlo simulation was applied to compare ZIF‐8 synthesis in GlyC‐best with ZIF‐8 synthesis in DMF by repeating the comparison for 1000 runs (Supporting Information section, Figure S7.5), applying the CED V1.11 method (single issue). In 36.5% of the evaluated cases, the Z‐GlyC‐best scenario demonstrates superior performance compared to Z‐DMF.

## 5 Conclusions

Two different routes based on the solvothermal synthesis of ZIF‐8 have been thoroughly assessed in a life cycle perspective. In detail, DMF‐base synthesis of ZIF‐8 was compared to an innovative method that involves the use of a biobased solvent, i.e., GlyC. The life cycle analysis covered all the steps from cradle‐to‐gate.

In summary, the environmental impacts related to the innovative scenario (Z‐GlyC‐base) were deeply investigated, and the main hotspots were identified, as they appeared as the one featured by the highest environmental impacts. The predominant contributor to the impacts is the energy required for the GlyC purification phase through distillation, accounting for the majority of the categories from approximately 36.4% up to 62.3% of the total outcomes, followed by DMC and glycerol. Consequently, a sensitivity analysis pointed out that by applying improvements to the process, (the Z‐GlyC‐best) scenario could be competitive with the synthesis carried out in DMF, especially given the urgency of replacing DMF in chemical processes, whereby possible.

We encourage the adoption of the LCA methodology to highlight the concept according to which the bare replacement of a toxic molecule (i.e., DMF) with a bioderived and safer one (i.e., GlyC) is not the only requirement responsible for determining the environmental sustainability of a process, which must be appropriately evaluated throughout its entire life cycle.

## Supporting Information

Additional supporting information can be found online in the Supporting Information section. **Supporitng** **Fig.** **S1.1:** ^1^H‐NMR spectrum of Glycerol carbonate (GlyC) (300 MHz, DMSO). **Supporting** **Fig.** **S1.2:** ^13^C‐NMR of GlyC (100 MHz, DMSO), δ: 155.2 (CO), 77.1 (CH), 65.9 (CH2), 60.6 (CH2). **Supporting** **Fig. S2.1:** Flowchart of DMF‐based Scenario (Z‐DMF). **Sup** **porting** **Fig**. **S6.1:** Contribution analysis expressed in cumulative single score through the ReCiPe EndPoint H approach for Z‐DMF. **Supporting** **Fig.** **S7.3:** Uncertainty analysis of 1 g ‘Z‐DMF' (A) minus 1 g ‘Z‐GlyC‐best' (B). Method: ReCiPe 2016 Endpoint (H) V1.05 / World (2010) H/A, confidence interval: 95%. **Supporting** **Fig.** **S7.4:** Uncertainty analysis of 1 g ‘Z‐DMF' (A) minus 1 g ‘Z‐GlyC‐best' (B). Method: ReCiPe 2016 Endpoint (H) V1.05 / World (2010) H/A, confidence interval: 95%. **Supporting** **Fig.** **S7.5:** Uncertainty analysis of Z‐DMF > Z‐GlyC‐best. **Supporting Table S2.1.1**: LCI of DMF‐based Scenario (Z‐DMF). **Supporting Table S2.1.2**: LCI of GlyC_baseline Scenario (Z‐GlyC‐base). **Supporting** **Table** **S4.1:** Energy requirement calculation for Z‐DMF scenario (*T*_lab_ = 19°C). **Supporting Table S4.2:** Energy requirement calculation for Z‐GlyC scenario (*T*_lab_ = 19°C). **Supporting** **Table** **S5.1.1:** LCIA of the Z‐DMF scenario ReCiPe 2016 Midpoint (H) V1.08/World (2010) H/A. **Supporting** **Table** **S5.1.2:** LCIA of the Z‐DMF scenario applying ReCiPe 2016 EndPoint (H) V1.08/World (2010) H/A. **Supporting** **Table** **S5.1.3:** LCIA of the Z‐DMF scenario applying Cumulative Energy Demand (CED) V1.11. **Supporting** **Table** **S5.2.1:** LCIA of the Z‐GlyC‐*base scenario* ReCiPe 2016 MidPoint (H) V1.08/World (2010) H/A. **Supporting** **Table** **S5.2.2:** LCIA of the Z‐GlyC‐*base scenario* ReCiPe 2016 EndPoint (H) V1.08/World (2010) H/A. **Supporting** **Table** **S5.2.3:** LCIA of the Z‐GlyC‐ *base scenario* Cumulative Energy Demand (CED) V1.11. **Supporting** **Table** **S7.1:** Pedigree Matrix value for both Z‐DMF and Z‐GlyC. **Supporting** **Table** **S7.2:** SD^2^ values for both Z‐DMF and Z‐GlyC.

## Funding

University of Salerno for research fund (ORSA234311); Italian Ministry of University and Research; HUN‐REN Hungarian Research Network and the National Research, Development and Innovation Office of Hungary (K146071).

## Conflicts of Interest

The authors declare no conflicts of interest.

## Supporting information

Supplementary Material

## Data Availability

The data supporting this article have been included as part of the Supplementary Information.
