# Supplementary material for: Life Cycle Assessment of Solvothermal Zeolitic Imidazolate Framework‐8 Synthesis: Is the Substitution of N,N‐Dimethylformamide with Glycerol Carbonate Environmentally Sustainable?
Source: ChemSusChem. 2025 Nov 16;18(24):e202502019. doi: 10.1002/cssc.202502019 (PMC12703451; doi:10.1002/cssc.202502019)
Supplement: Supplementary file 1 — Supplementary Material [file CSSC-18-e202502019-s001.pdf]

Supporting Information (SI)

Life Cycle Assessment of Solvothermal ZIF-8 Synthesis: Is the Substitution of DMF with Glycerol Carbonate Environmentally Sustainable?

Alessandra Sessa,[a] Eleonora Rossi,[b] Prisco Prete,[a] Fabrizio Passarini,[b] Masaki Itatani,[c] Federico Rossi,[d] Istvan Lagzi,[e,f] Pierandrea Lo Nostro,[g] Daniele Cespi,*[b] Raffaele Cucciniello, *[a]

[a]*Department of Chemistry and Biology "Adolfo Zambelli", Salerno University, Via Giovanni Paolo II 132, 84084 Fisciano, SA, Italy email: rcucciniello@unisa.it*
[b]*Industrial Chemistry Department "Toso Montanari", Alma Mater - Università di Bologna, Via Piero Gobetti, 85, 40129 Bologna, BO, Italy, email: daniele.cespi2@unibo.it*
[c] *Department of Chemistry, Faculty of Science, Hokkaido University, Sapporo, Hokkaido 060-0810, Japan*
[d]*Department of Physical Sciences, Earth and Environment, University of Siena, 53100 Siena, Italy*
[e] *Department of Physics, Institute of Physics, Budapest University of Technology and Economics, H-1111 Budapest, Hungary*
[f] *HUN-REN–BME, Condensed Matter Physics Research Group, Budapest University of Technology and Economics, H-1111 Budapest, Hungary*
[g] *Department of Chemistry "Ugo Schiff", University of Florence, 50019 Sesto Fiorentino (Firenze), Italy*

Corresponding authors: rcucciniello@unisa.it ; daniele.cespi2@unibo.it

S1. Glycerol carbonate synthesis and characterization

GlyC was synthesized through the transcarbonation of glycerol with dimethyl carbonate (DMC) using Na_2CO_3 as a catalyst. 517.07 g of DMC, 176.07 g of glycerol, and 0.61g of Na_2CO_3 were transferred into a 2L three-necked flask equipped with a magnetic stirrer (300 rpm) and subjected to heating at 100°C for 2h. Subsequently, the product was filtered to remove the catalyst, and the product was distilled under reduced pressure to remove the formed methanol and the excess of DMC. GlyC was obtained as a viscous liquid, detected through ^{1}H-NMR and ^{13}C-NMR (Bruker AVANCE – 300 MHz using deuterated DMSO as the solvent), and the spectra are shown in Figure S1.1 and S1.2

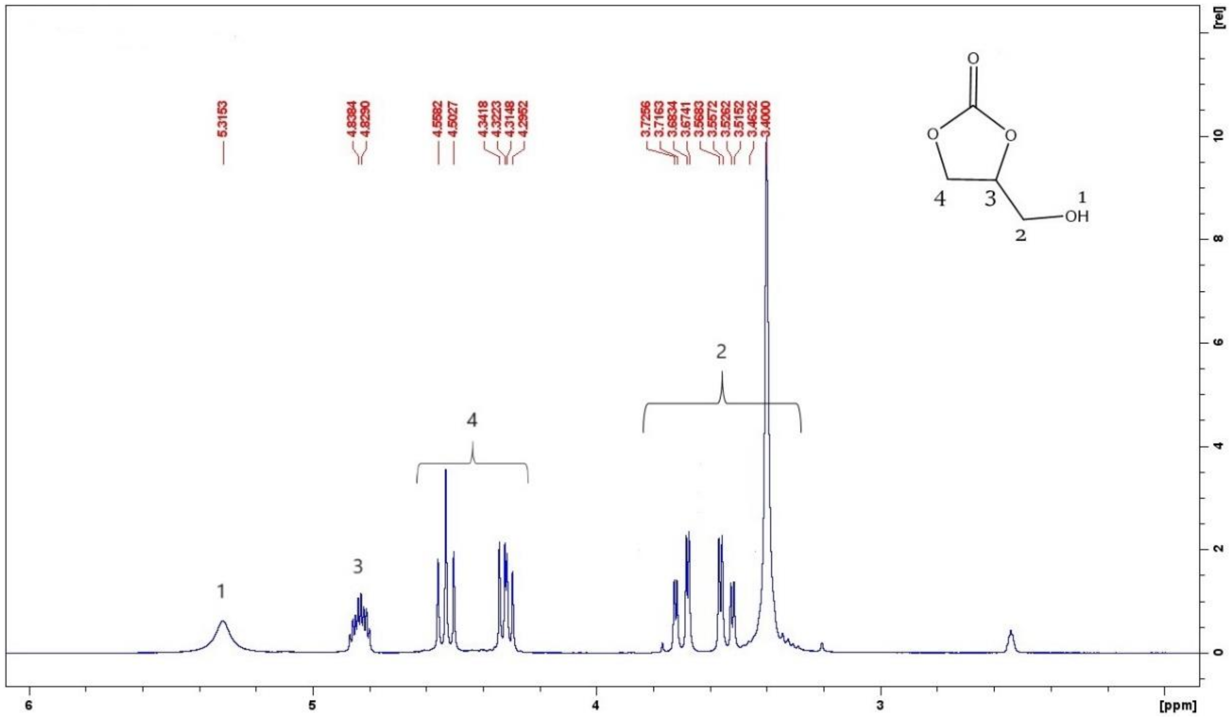

Figure S1.1 ^{1}H-NMR spectrum of Glycerol carbonate (GlyC) (300 MHz, DMSO).

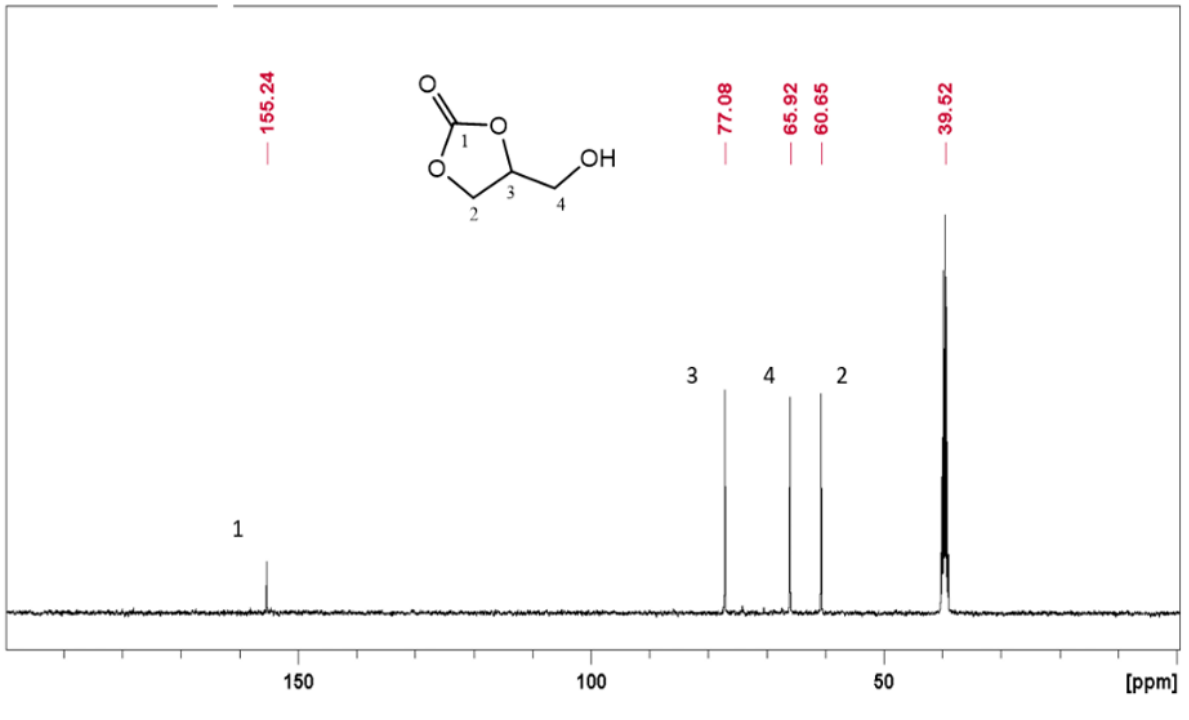

Figure S1.2. ^{13}C-NMR of GlyC (100 MHz, DMSO), δ: 155.2 (CO), 77.1 (CH), 65.9 (CH2), 60.6 (CH2).

S2. Life Cycle Inventory (LCI)

S2.1 LCI of the main scenarios

Table S2.1.1 LCI of DMF-based Scenario (Z-DMF)

Item	Ecoinvent process	Type of process	Quantity	Unit of measurement
Zn $(NO_3)_2 \cdot 6H_2O$	*_zinc nitrate 6 H_2O**	INPUT	3.28	g
2-methylimidazole	*_2-Methylimidazole**	INPUT	7.24	g
DMF	*N, N-dimethylformamide {GLO} | market for | APOS, U*	INPUT	51	g
Energy- ZIF-8 synthesis	*Electricity, medium voltage {GLO}| market group for | APOS, U*	INPUT	10.25	kWh
MeOH	*Methanol {GLO}| market for | APOS, U*	INPUT	20	g
Energy (Centrifugation)	*Electricity, medium voltage {GLO}| market group for | APOS, U*	INPUT	0.30	kWh
Energy (Drying)	*Electricity, medium voltage {GLO}| market group for | APOS, U*	INPUT	0.08	kWh
Waste to incineration	*spent solvent mixture {Europe without Switzerland} | treatment of spent solvent mixture, hazardous waste incineration, with energy recovery | APOS, U*	OUTPUT	60.39	g

Table S2.1.2 LCI of GlyC_baseline Scenario (Z-GlyC-base).

Item	Ecoinvent process	Type of process	Quantity	Unit of measurement
glycerol (RO)	*Glycerine {Europe without Switzerland} | esterification of rape oil | APOS, U*	INPUT	176.07	g
DMC	*Dimethyl carbonate {GLO}| market for dimethyl carbonate | APOS, U*	INPUT	517.07	g
Na_2CO_3	*Soda ash, dense {GLO}| market for | APOS, U*	INPUT	0.61	g
energy for GlyC synthesis	*Electricity, medium voltage {GLO}| market group for | APOS, U*	INPUT	0.33	kWh
energy for GlyC distillation	*Electricity, medium voltage {GLO}| market group for | APOS, U*	INPUT	2.75	kWh
Waste to incineration *MeOH + DMC*	*spent solvent mixture {Europe without Switzerland} | treatment of spent solvent mixture, hazardous waste incineration, with energy recovery | APOS, U*	OUTPUT (waste)	(61+176)	g
Zn $(NO_3)_2 \cdot 6H_2O$	*_zinc nitrate 6 H2O (ZIF-8)*	INPUT	1.82	g
2-methylimidazole	*_2-Methylimidazole*	INPUT	1.36	g

Energy for ZIF-8 synthesis	*Electricity, medium voltage {GLO}| market group for | APOS, U*	INPUT	5.71	kWh
Energy (ultrasonic)	*Electricity, medium voltage {GLO}| market group for | APOS, U*	INPUT	0.15	kWh
MeOH	*Methanol {GLO}| market for | APOS, U*	INPUT	20	g
Energy (centrifugation)	*Electricity, medium voltage {GLO}| market group for | APOS, U*	INOPUT	0.30	kWh
Waste to incineration	*spent solvent mixture {Europe without Switzerland} | treatment of spent solvent mixture, hazardous waste incineration, with energy recovery | APOS, U*	OUTPUT (waste)	204.39	g

S2.2 GlyC-based optimized scenarios

Mass balance of GlyC_baseline Scenario (Z-GlyC-*wco*): identical to Z-GlyC-base, it differs from this only for the origin of glycerol, Glycerine {FR}| treatment of waste cooking oil, purified, esterification | APOS, U, replacing "*Glycerine {Europe without Switzerland} | esterification of rape oil | APOS, U*"

Mass balance of GlyC_baseline Scenario (Z-GlyC-*avp*): in this scenario both *Dimethyl carbonate {GLO}| market for dimethyl carbonate | APOS, U and Methanol {GLO}| market for | APOS, U appear as "**avoided product**"* in the model.

Mass balance of GlyC_baseline Scenario (Z-GlyC-*best*): the improvements applied to Z-GlyC-wco and to Z-GlyC-avp were incorporated into this model.

The models for *_zinc nitrate, _zinc acetate, _2-methylimidazole* were designed as follows:

- *_2-methylimidazole:* It is prepared by condensation of glyoxal, ammonia, and acetaldehyde, nitration gives the 5-nitro derivative. Starting from "*Imidazole {RER}| production| APOS, U*" formaldehyde was replaced by acetaldehyde and ammonia was replaced by methylamine and calculations (all reagents' quantities) were adjusted to be in agreement with the stoichiometry of the new reaction:

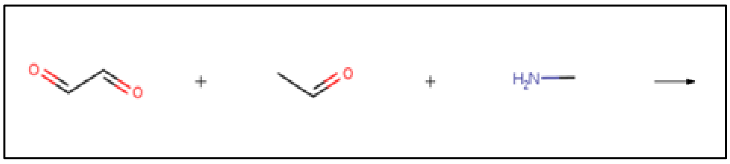

a) 0.4644kg of formaldehyde (MW=30.031 g/mol) → 15,46 mol

MW (acetaldehyde) = 44.05 g /mol → *w*(acetaldehyde) = 15.46 mol * MW = 0.681 kg.

b) 0.5263 kg of NH_3 (MW = 17.031 g/mol) → *n* = 30.9 mol

MW CH_3NH_2 = 31.05 g/mol, w(CH_3NH_2) = 0.959 kg

Item	Ecoinvent process	Type of process	Quantity	Unit of measurement
2-Methylimidazole	New	Product	1	kg
Water	Water, cooling, unspecified, natural origin, RER	INPUT	0.024	m^3
Methylamine	Methylamine {RER}, market for methylamine | APOS, U	INPUT	0.959	kg
Chemical factory, organics {RER}	Construction | APOS, U	INPUT	$4x10^{-10}$	p
Acetaldehyde	Acetaldehyde {GLO} market for | APOS, U	INPUT	0.681	kg
Glyoxal	Glyoxal {RER}| market for glyoxal | APOS, U	INPUT	0.898	kg

- *_zinc nitrate hexahydrate:* $ZnO + 2 HNO_3 \rightarrow Zn(NO_3)_2 + H_2O$

Item	Ecoinvent process	Type of process	Quantity	Unit of measurement
zinc nitrate hexahydrate		product	*1*	*g*
Zinc oxide	zinc oxide {GLO} | market for | APOS, U	INPUT	*0.4230*	*g*
Nitric acid	nitric acid, without water, in 50% solution state {GLO} | market for | APOS, U	INPUT	*0.665*	*g*
Water	Water, deionized {EU-Switzerland}, water production, deionized | APOS, U	INPUT	*0.363*	*g*

- *_zinc_acetate diihydrate:* **Current Patent Assignee:** RU2483056C2, 2011

Item	Ecoinvent process	Type of process	Quantity	Unit of measurement
zinc acetate dihydrate		product	*1*	*g*
Zinc oxide	*zinc oxide {GLO} | market for | APOS, U*	INPUT	*0.370*	*g*
Acetic acid	*nitric acid, without water, in 98% solution state {GLO} | market for | APOS, U*	INPUT	*0.546*	*g*
Water	*Water, deionized {EU-Switzerland}, water production, deionized | APOS, U*	INPUT	*0.164*	*g*

S3. Z-DMF scenario

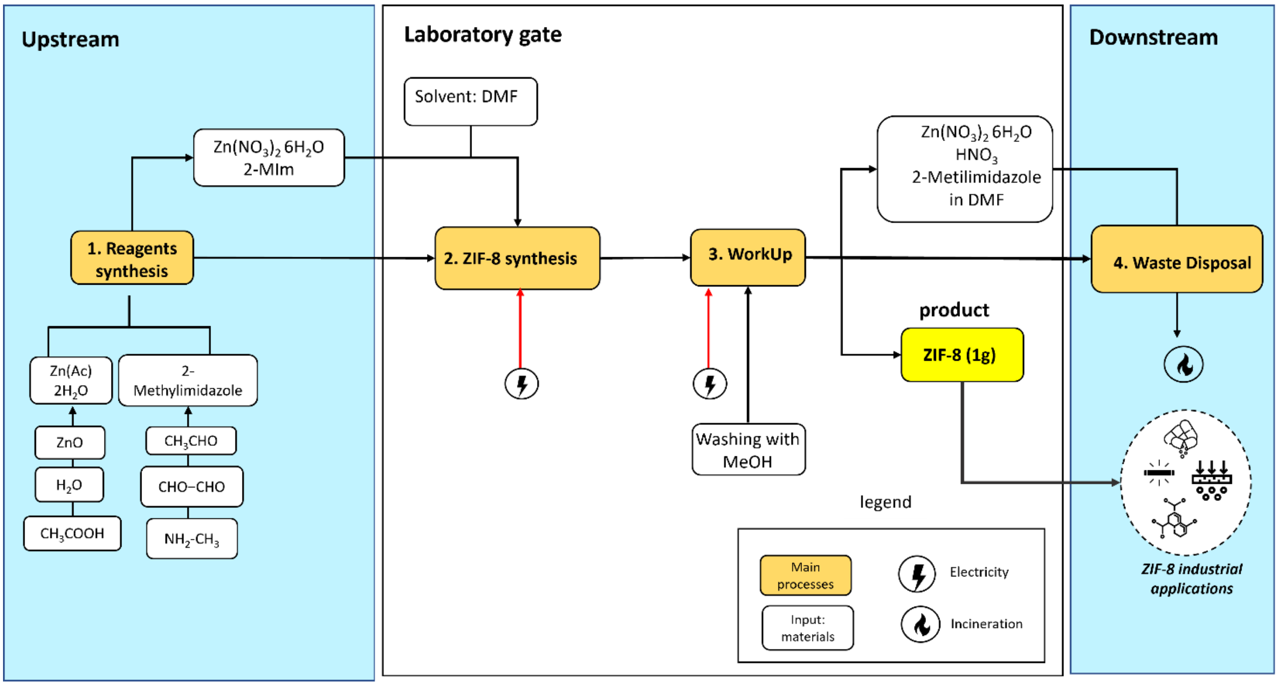

Figure S2.1 Flowchart of DMF-based Scenario (Z-DMF).

S4. Energy consumption data sheet

For all calculations, standard laboratory instrument power has been considered. Also, the following models for the instrument are: Hermle Labortechnik(centrifuge), Heidolph -MR Hei-Standard (heating plate), Leroy Somer (vacuum pump), DERUI DR-MH60 (Ultrasonic cleaner), GLOBEINSTRUMENTS (Oven).

For temperature allocation: power and time of usage were multiplied by the factor $\Delta t/\Delta t_{max}$ = ($T_{reaction}$-T_{ext})/(T_{max}-T_{ext}), where T_{ext}= $T_{laboratory}$= 19°C ca.

Table S4.1 Energy requirement calculation for Z-DMF scenario (T_{lab} = 19 °C).

**Convenction oven**	**Unit**	
Power	0.75	kW
Tmax	250	°C
**ZIF-8 synthesis**		
Treaction	140	°C
treaction	24	h
Work-up		
Treaction	120	°C
treaction	12	h

ΔT/Δtmax	0.57	
Speedmax	0.18	
$Energy_{(Synthesis)}$	10.25	kWh
$Energy_{(Essicc.)}$	0.08	kWh

Table S4.2 Energy requirement calculation for Z-GlyC scenario (T_{lab} = 19 °C)

**Parameter**	Heating Plate (Heidolph MR Hei-Standard)	Vacuum pump (Leroy Somer)	Centrifuge (Hermle Labortechnik)	Ultrasonic (DERUI DR-MH60)
**Power**	0.825 kW	0.55 kW	0.58 kW	0.480 kW
**Max Temperature (Tmax)**	300 °C			
**Max Speed (Speedmax)**	1400 rpm			

**Parameter**	**GlyC-synthesis**	**GlyC Distillation**	**Work Up ZIF-8**	**Precursors' solutions**
**T reaction (°C)**	75 °C	65°C		RT(Zn), 80°C (2-MIm)
**Time**	2 h	5h	20 min (tot.)	2 h(Zn), 1.5h(2-MIm)
**Speed**	250 rpm		5000 rpm	
**ΔtT/ΔTmax**	0.20	0.20		
**Speed/Speed Max**	0.18	0.18	0.52	
**Energy**	0.33 kWh	2.75 kWh	0.30 kWh	0.378 kWh (Zn), 0.72(2-MIm)

ZIF-8 Synthesis

**Parameter**	$T_{reaction}$	**Speed**	**Time**	$\Delta T/T_{max}$	**Speed/Speed** m_{ax}	**Energy**
**Value**	100 °C	250 rpm	24 h	0.29	0.18	5.71 kWh

S5. Life Cycle Impact Assessment (LCIA)

Table S5.1.1 LCIA of the Z-DMF scenario ReCiPe 2016 Midpoint (H) V1.08 / World (2010) H/A.

**Impact category**	**Unit**	**2-MIm (g)**	**Zn $(NO_3)_2$ (g)**	**DMF (g)**	**Electricity for mixing (kWh)**	**MeOH - Washing step (g)**	**Energy - Centrifugation (kWh)**	**Energy - Essiccation (kWh)**	**Waste (to incineration) (g)**	**total**
**Global warming**	*kg CO_2 eq*	4.87 x10^{-2}	6.40 x10^{-3}	1.56 x10^{-1}	7.66	1.39 x10^{-2}	2.25 x10^{-1}	5.78 x10^{-2}	1.19 x10^{-1}	7.96

**Stratospheric ozone depletion**	*kg CFC11 eq*	1.29 x10^{-8}	1.13 x10^{-7}	6.05 x10^{-8}	3.18 x10^{-6}	7.35 x10^{-9}	9.34 x10^{-8}	2.40 x10^{-8}	3.16 x10^{-8}	3.30 x10^{-6}
**Ionizing radiation**	*kBq Co-60eq*	4.62 x10^{-3}	1.20 x10^{-4}	9.41 x10^{-3}	9.27 x10^{-1}	2.23 x10^{-4}	2.72 x10^{-2}	6.99 x10^{-3}	5.83 x10^{-4}	9.45 x10^{-1}
**Ozone formation, Human health**	*kg NOx eq*	7.60 x10^{-5}	9.40 x10^{-6}	2.98 x10^{-4}	1.67 x10^{-2}	2.58 x10^{-5}	4.90 x10^{-4}	1.26 x10^{-4}	4.79 x10^{-5}	1.68 x10^{-2}
**Fine particulate matter formation**	*kg PM2.5 eq*	4.79 x10^{-5}	5.44 x10^{-6}	2.08 x10^{-4}	1.67 x10^{-2}	1.30 x10^{-5}	4.91 x10^{-4}	1.26 x10^{-4}	2.01 x10^{-5}	1.69 x10^{-2}
**Ozone formation, Terrestrial ecosystems**	*kg NOx eq*	7.74 x10^{-5}	9.56 x10^{-6}	3.03 x10^{-4}	1.68 x10^{-2}	2.63 x10^{-5}	4.94 x10^{-4}	1.27 x10^{-4}	4.79 x10^{-5}	1.68 x10^{-2}
**Terrestrial acidification**	*kg SO_2 eq*	1.44 x10^{-4}	2.05 x10^{-5}	4.92 x10^{-4}	2.57 x10^{-2}	3.57 x10^{-5}	7.54 x10^{-4}	1.94 x10^{-4}	4.46 x10^{-5}	2.60 x10^{-2}
**Freshwater eutrophication**	*kg P eq*	1.60 x10^{-5}	9.42 x10^{-7}	5.73 x10^{-5}	3.78 x10^{-3}	1.65 x10^{-6}	1.11 x10^{-4}	2.85 x10^{-5}	2.42 x10^{-5}	3.88 x10^{-3}
**Marine eutrophication**	*kg N eq*	2.64 x10^{-5}	7.16 x10^{-8}	1.56 x10^{-4}	2.78 x10^{-4}	1.32 x10^{-7}	8.15 x10^{-6}	2.10 x10^{-6}	2.29 x10^{-6}	4.55 x10^{-4}
**Terrestrial ecotoxicity**	*kg 1,4-DCB*	4.63 x10^{-3}	8.63 x10^{-2}	7.83 x10^{-2}	5.61	4.25 x10^{-4}	1.65 x10^{-1}	4.23 x10^{-2}	2.37 x10^{-3}	1.24
**Freshwater ecotoxicity**	*kg 1,4-DCB*	6.09 x10^{-5}	2.44 x10^{-4}	3.58 x10^{-4}	4.35 x10^{-1}	1.30 x10^{-5}	1.28 x10^{-2}	3.28 x10^{-3}	3.32 x10^{-5}	1.33 x10^{-2}
**Marine ecotoxicity**	*kg 1,4-DCB*	6.54 x10^{-5}	3.75 x10^{-4}	2.76 x10^{-4}	5.48 x10^{-1}	1.59 x10^{-5}	1.61 x10^{-2}	4.13 x10^{-3}	4.82 x10^{-5}	1.92 x10^{-2}
**Human carcinogenic toxicity**	*kg 1,4-DCB*	4.86 x10^{-4}	3.41 x10^{-4}	2.43 x10^{-5}	3.94 x10^{-1}	3.68 x10^{-6}	1.16 x10^{-2}	2.97 x10^{-3}	1.88 x10^{-5}	1.45 x10^{-3}
**Human non-carcinogenic toxicity**	*kg 1,4-DCB*	3.90 x10^{-4}	8.95 x10^{-3}	1.44 x10^{-3}	6.53	7.20 x10^{-5}	1.92 x10^{-1}	4.92 x10^{-2}	1.64 x10^{-4}	6.06 x10^{-2}
**Land use**	*m^2a crop eq*	3.25 x10^{-3}	2.40 x10^{-4}	9.36 x10^{-3}	3.22 x10^{-1}	3.25 x10^{-4}	9.44 x10^{-3}	2.43 x10^{-3}	5.45 x10^{-4}	3.40 x10^{-1}
**Mineral resource scarcity**	*kg Cu eq*	1.21 x10^{-4}	1.93 x10^{-5}	4.27 x10^{-4}	7.84 x10^{-3}	1.87 x10^{-5}	2.30 x10^{-5}	5.91 x10^{-5}	3.52 x10^{-5}	5.10 x10^{-3}
**Fossil resource scarcity**	*kg oil eq*	2.57 x10^{-2}	9.74 x10^{-4}	8.10 x10^{-2}	1.92	1.51 x10^{-2}	5.64 x10^{-2}	1.45 x10^{-2}	3.85 x10^{-3}	2.04
**Water consumption**	*m^3*	9.09 x10^{-4}	4.39 x10^{-5}	1.97 x10^{-3}	6.09 x10^{-2}	8.45 x10^{-5}	1.79 x10^{-3}	4.59 x10^{-4}	1.60 x10^{-4}	6.05 x10^{-2}

Table S5.1.2 LCIA of the Z-DMF scenario applying ReCiPe 2016 EndPoint (H) V1.08 / World (2010) H/A.

Damage-oriented category	Unit	2-MIM (g)	Zn $(NO_3)_2$ (g)	DMF (g)	Electricity for mixing (kWh)	MeOH – washing step (g)	Energy – Centrifugation step (kWh)	Waste (to incineration) (g)	total
**Total**	mPts	1.70	2.21 x10^{-1}	6.01	4.33 x10^{1}	4.52 x10^{-1}	5.38	2.26	3.56 x10^{-2}
**Human health**	mPts	1.57	2.12 x10^{-1}	5.62	4.18 x10^{1}	4.00 x10^{-1}	5.20	2.15	3.44 x10^{-2}
**Ecosystem**	mPts	6.10 x10^{-2}	7.55 x10^{-3}	1.92 x10^{-1}	1.16	1.46 x10^{-2}	1.45 x10^{-1}	9.94 x10^{-2}	9.70
**Resources**	mPts	6.60 x10^{-2}	2.07 x10^{-3}	1.95 x10^{-1}	3.12 x10^{-1}	3.81 x10^{-2}	3.89 x10^{-2}	1.02 x10^{-2}	2.82

Values for energy (exsiccation step) appear to be negligible (=0).

Table S5.1.3. LCIA of the Z-DMF scenario applying Cumulative Energy Demand (CED) V1.11

Impact category	Unit	2-MIm(g)	$Zn(NO_3)_2$ (g)	DMF (g)	En. Mixing (kWh)	MeOH (washing step) (g)	Energy – Centrifugation (kWh)	waste (to incineration) (g)
**Total**	mPts	1.31	3.71 x10^{-2}	3.99	1.18 x10^{-2}	6.96 x10^{-1}	3.47	1.93 x10^{-1}
**Non renewable, fossil**	mPts	1.17	3.41 x10^{-2}	3.70	8.83 x10^{1}	6.89 x10^{-1}	2.59	1.76 x10^{-1}
**Non-renewable, nuclear**	mPts	9.82 x10^{-2}	1.69 x10^{-3}	1.68 x10^{-1}	1.71 x10^{1}	3.97 x10^{-3}	5.03 x10^{-1}	9.87 x10^{-3}
**Non-renewable, biomass**	mPts	1.63 x10^{-5}	2.87 x10^{-6}	6.75 x10^{-5}	8.22 x10^{-3}	2.38 x10^{-6}	2.41 x10^{-4}	6.94 x10^{-6}
**Renewable, biomass**	mPts	1.16 x10^{-2}	4.92 x10^{-4}	3.39 x10^{-2}	2.08	5.30 x10^{-4}	6.10 x10^{-2}	1.84 x10^{-3}
**Renewable, wind, solar, geothe**	mPts	1.05 x10^{-2}	1.94 x10^{-4}	2.19 x10^{-2}	3.13	5.03 x10^{-4}	9.18 x10^{-2}	1.34 x10^{-3}
**Renewable, water**	mPts	1.89 x10^{-2}	6.29 x10^{-4}	6.06 x10^{-2}	7.73	1.77 x10^{-3}	2.27x10^{-1}	3.95 x10^{-3}

Values for energy (exsiccation step) appear to be negligible (=0).

Table S5.2.1 LCIA of the Z-GlyC-*base* scenario ReCiPe 2016 MidPoint (H) V1.08 / World (2010) H/A.

Impact category	Unit	Glycerol_RO (g)	DMC (g)	Na_2CO_3 (g)	Electricity for mixing (kWh)	Energy Dist (kWh)	Waste to incineration (g)	Zn $(OAc)_2$ (g)	Washing step	2Mim	Energy (Ultras) (kWh)	Energy (Ultras) (kWh)	NaOH(g)	Electricity for mixing (kWh)	Energy Centrifugation (kWh)	Incineration
**GWP**	kg CO2 eq	3.03 x10^{-1}	1.17	7.79 x10^{-4}	2.46 x10^{-1}	2.06	4.68 x10^{-1}	2.24 x10^{-3}	6.11 x10^{-2}	9.15 x10^{-3}	7.17 x10^{-2}	3.77 x10^{-2}	4.21 x10^{-4}	4.27	2.25 x10^{-1}	4.04 x10^{-1}
**SOD**	kg CFC11 eq	4.75 x10^{-6}	3.10 x10^{-2}	2.02 x10^{-10}	1.02 x10^{-7}	8.54 x10^{-7}	1.24 x10^{-7}	1.07 x10^{-9}	2.37 x10^{-8}	2.43 x10^{-9}	2.98 x10^{-8}	1.56 x10^{-8}	4.53 x10^{-10}	1.77 x10^{-6}	9.34 x10^{-8}	1.07 x10^{-2}
**IR**	kBq Co-60eq	1.33 x10^{-2}	8.35 x10^{-2}	3.65 x10^{-5}	2.97 x10^{-2}	2.49 x10^{-1}	2.29 x10^{-3}	1.60 x10^{-4}	3.69 x10^{-3}	8.69 x10^{-4}	8.68 x10^{-3}	4.56 x10^{-3}	4.66 x10^{-5}	5.16 x10^{-1}	2.72 x10^{-2}	1.97 x10^{-3}
**OF_HH**	kg NOx eq	9.32 x10^{-4}	2.44 x10^{-3}	1.47 x10^{-6}	5.35 x10^{-4}	4.48 x10^{-3}	1.88 x10^{-4}	5.56 x10^{-6}	1.17 x10^{-4}	1.43 x10^{-5}	1.56 x10^{-4}	8.21 x10^{-5}	1.09 x10^{-6}	9.29 x10^{-3}	4.90 x10^{-4}	1.62 x10^{-4}

**FPMF**	kg PM2.5 eq	7.88 x10^{-4}	1.39 x10^{-3}	1.05 x10^{-6}	5.37 x10^{-4}	4.49 x10^{-3}	7.90 x10^{-5}	3.62 x10^{-6}	8.17 x10^{-5}	9.00 x10^{-6}	1.57 x10^{-4}	8.23 x10^{-5}	9.08 x10^{-7}	9.32 x10^{-3}	4.91 x10^{-4}	6.81 x10^{-5}
**OF_TE**	kg NOx eq	9.38 x10^{-4}	2.57 x10^{-3}	1.48 x10^{-6}	5.40 x10^{-4}	4.51 x10^{-3}	1.88 x10^{-4}	5.87 x10^{-6}	1.19 x10^{-4}	1.46 x10^{-5}	1.58 x10^{-4}	8.27 x10^{-5}	1.09 x10^{-6}	9.37 x10^{-3}	4.94 x10^{-4}	1.62 x10^{-4}
**TA**	kg SO2 eq	4.47 x10^{-3}	3.04 x10^{-3}	2.40 x10^{-6}	8.24 x10^{-4}	6.89 x10^{-3}	1.75 x10^{-4}	7.36 x10^{-6}	1.93 x10^{-4}	2.70 x10^{-5}	2.41 x10^{-4}	1.26 x10^{-4}	1.53 x10^{-6}	1.43 x10^{-2}	7.54 x10^{-4}	1.51 x10^{-4}
**FE**	kg P eq	9.60 x10^{-5}	3.50 x10^{-4}	2.49 x10^{-7}	1.21 x10^{-4}	1.01 x10^{-3}	9.48 x10^{-5}	7.08 x10^{-7}	2.25 x10^{-5}	3.01 x10^{-6}	3.54 x10^{-5}	1.86 x10^{-5}	2.14 x10^{-7}	2.10 x10^{-3}	1.11 x10^{-4}	8.18 x10^{-5}
**ME**	kg N eq	1.77 x10^{-3}	3.19 x10^{-5}	5.44 x10^{-7}	8.91 x10^{-6}	7.45 x10^{-5}	9.00 x10^{-6}	5.47 x10^{-8}	6.11 x10^{-5}	4.96x10^{-6}	2.60 x10^{-6}	1.37 x10^{-6}	2.21 x10^{-8}	1.55 x10^{-4}	8.15 x10^{-6}	7.76 x10^{-6}
**TET**	kg 1,4-DCB	2.79 x10^{-2}	1.04	5.47 x10^{-4}	1.80 x10^{-1}	1.51	9.31 x10^{-3}	4.31 x10^{-2}	3.07 x10^{-2}	8.71 x10^{-4}	5.26 x10^{-2}	2.76 x10^{-2}	7.24 x10^{-5}	3.13	1.65 x10^{-1}	8.03 x10^{-3}
**FET**	kg 1,4-DCB	8.86 x10^{-4}	1.64 x10^{-3}	1.12 x10^{-6}	1.40 x10^{-2}	1.17 x10^{-1}	1.30 x10^{-4}	1.32 x10^{-4}	1.40 x10^{-4}	1.14 x10^{-5}	4.07 x10^{-3}	2.14 x10^{-3}	8.23 x10^{-7}	2.42 x10^{-1}	1.28 x10^{-2}	1.12 x10^{-4}
**MET**	kg 1,4-DCB	8.32 x10^{-4}	2.14 x10^{-3}	1.53 x10^{-6}	1.76 x10^{-2}	1.47 x10^{-1}	1.89 x10^{-4}	2.01 x10^{-4}	1.08 x10^{-4}	1.23 x10^{-5}	5.13 x10^{-3}	2.69 x10^{-3}	1.20 x10^{-6}	3.05 x10^{-1}	1.61 x10^{-2}	1.63 x10^{-4}
**HCT**	kg 1,4-DCB	4.60 x10^{-5}	7.22 x10^{-4}	1.52 x10^{-7}	1.26 x10^{-2}	1.06 x10^{-1}	7.37 x10^{-5}	2.01 x10^{-4}	9.52 x10^{-6}	9.13 x10^{-5}	3.69 x10^{-3}	1.94 x10^{-3}	1.53 x10^{-7}	2.19 x10^{-1}	1.16 x10^{-2}	6.36 x10^{-5}
**HNCT**	kg 1,4-DCB	3.03 x10^{-3}	1.40 x10^{-2}	9.79 x10^{-6}	2.09 x10^{-1}	1.75	6.45 x10^{-4}	4.57 x10^{-3}	5.65 x10^{-4}	7.33 x10^{-5}	6.12 x10^{-2}	3.21 x10^{-2}	6.45 x10^{-6}	3.64	1.92 x10^{-1}	5.56 x10^{-4}
**LU**	m2a crop eq	1.00	7.70 x10^{-2}	5.07 x10^{-5}	1.03 x10^{-2}	8.63 x10^{-2}	2.14 x10^{-3}	1.48 x10^{-4}	3.67 x10^{-3}	6.10 x10^{-4}	3.01 x10^{-3}	1.58 x10^{-3}	3.04 x10^{-5}	1.79 x10^{-1}	9.44 x10^{-3}	1.85 x10^{-3}
**MRS**	kg Cu eq	1.13 x10^{-3}	4.25 x10^{-3}	3.27 x10^{-6}	2.52 x10^{-4}	2.10 x10^{-3}	1.38 x10^{-4}	8.30 x10^{-6}	1.67 x10^{-4}	2.28 x10^{-5}	7.34 x10^{-5}	3.86 x10^{-5}	1.34 x10^{-6}	4.37 x10^{-3}	2.30 x10^{-4}	1.19 x10^{-4}
**FRS**	kg oil eq	6.40 x10^{-2}	5.81 x10^{-1}	1.94 x10^{-4}	6.17 x10^{-2}	5.16 x10^{-1}	1.51 x10^{-2}	1.17 x10^{-3}	3.18 x10^{-2}	4.83 x10^{-3}	1.80 x10^{-2}	9.46 x10^{-3}	1.05 x10^{-4}	1.07	5.64 x10^{-2}	1.30 x10^{-2}
**WC**	m3	1.50 x10^{-2}	1.13 x10^{-2}	1.41 x10^{-5}	1.95 x10^{-3}	1.63 x10^{-2}	6.29 x10^{-4}	4.72 x10^{-5}	7.73 x10^{-4}	1.71 x10^{-4}	5.70 x10^{-4}	2.99 x10^{-4}	1.12 x10^{-5}	3.39 x10^{-2}	1.79 x10^{-3}	5.42 x10^{-4}

Table S5.2.2 LCIA of the Z-GlyC-*base* scenario ReCiPe 2016 EndPoint (H) V1.08 / World (2010) H/A.

Damage category	Unit	glycerol_ RO (g)	DMC (g)	Na_2CO_3 (g)	Electricity for mixing(kWh)	Energy Dist(kWh)	Waste to incineration	$Zn(OAc)_2$ (g)	2-mim (g)	EnergyUltras(kWh)	Energy - Uktras(kWh)	Washing step	Energy heating plate (kWh)	Energy Centrifugation(kWh)	Incineration
**Human health**	mPts	1.82 x10^{1}	4.18 x10^{1}	3.00 x10^{-2}	1.10 x10^{1}	9.17 x10^{1}	8.45	1.03 x10^{-1}	2.96 x10^{-1}	3.20	1.68	2.21	1.90 x10^{2}	1.00 x10^{1}	7.29
**Ecosystem**	mPts	2.96	1.45	9.95 x10^{-4}	3.05 x10^{1}	2.55	3.90 x10^{1}	3.12 x10^{-3}	1.15 x10^{-2}	8.91 x10^{-2}	4.68 x10^{-2}	7.52 x10^{-2}	5.30	2.79 x10^{1}	3.36 x10^{1}
**Resources**	mPts	1.60 x10^{-1}	1.48	3.73 x10^{-4}	8.19 x10^{-2}	6.85 x10^{-1}	3.99 x10^{-2}	2.90 x10^{-3}	1.24 x10^{-2}	2.39 x10^{-2}	1.26 x10^{-2}	7.63 x10^{-2}	1.42	7.49 x10^{-2}	3.45 x10^{-2}

Table S5.2.3 LCIA of the Z-GlyC- *base* scenario Cumulative Energy Demand (CED) V1.11

Impact category	Unit	Glycerol RO (g)	DMC (g)	Na2CO3 (g)	Electricity for mixing (kWh)	Electricity for distillation(kWh)	Mixture to incineration	zinc acetate hexahydrate (g)	2-methylimidazole (g)	Energy sonication 2mim(kWh)	Energy sonication (kWh)	Energy heating plate (kWh)	Energy Centrifugation(kWh)	Incineration
**Non renewable, fossil**	MJ	2.92	2.65 x10^{1}	8.89 x10^{-3}	2.83	2.37 x10^{1}	6.92 x10^{-1}	6.41 x10^{-2}	2.20 x10^{-1}	8.27 x10^{-1}	4.34 x10^{-1}	4.92 x10^{1}	2.59	5.97 x10^{-1}
**Non-renewable, nuclear**	MJ	2.42 x10^{-1}	1.86	6.72 x10^{-4}	5.50 x10^{-1}	4.60	3.87 x10^{-2}	3.39 x10^{-3}	1.85 x10^{-2}	1.61 x10^{-1}	8.43 x10^{-2}	9.55	5.03 x10^{-1}	3.34 x10^{-2}
**Non-renewable, biomass**	MJ	8.80 x10^{-4}	5.27 x10^{-4}	3.71 x10^{-7}	2.64 x10^{-4}	2.20 x10^{-3}	2.72 x10^{-5}	2.75 x10^{-6}	3.06 x10^{-6}	7.69 x10^{-5}	4.04 x10^{-5}	4.57 x10^{-3}	2.41 x10^{-4}	2.35 x10^{-5}
**Renewable, biomass**	MJ	7.57	2.99 x10^{-1}	2.04 x10^{-4}	6.67 x10^{-2}	5.58 x10^{-1}	7.22 x10^{-3}	7.79 x10^{-4}	2.17 x10^{-3}	1.95 x10^{-2}	1.02 x10^{-2}	1.16	6.10 x10^{-2}	6.22 x10^{-3}
**Renewable, wind, solar, geothe**	MJ	2.30 x10^{-2}	1.95 x10^{-1}	8.59 x10^{-1}	1.00 x10^{-1}	8.40 x10^{-1}	5.24 x10^{-3}	4.45 x10^{-4}	1.97 x10^{-3}	2.93 x10^{-2}	1.54 x10^{-2}	1.74	9.18 x10^{-2}	4.52 x10^{-3}
**Renewable, water**	MJ	1.03 x10^{-1}	5.08 x10^{-1}	3.01 x10^{-4}	2.48 x10^{-1}	2.07	1.55 x10^{-2}	1.39 x10^{-3}	3.56 x10^{-3}	7.24 x10^{-2}	3.80 x10^{-2}	4.31	2.27 x10^{-1}	1.34 x10^{-2}

S6. Contribution analysis for Z-DMF

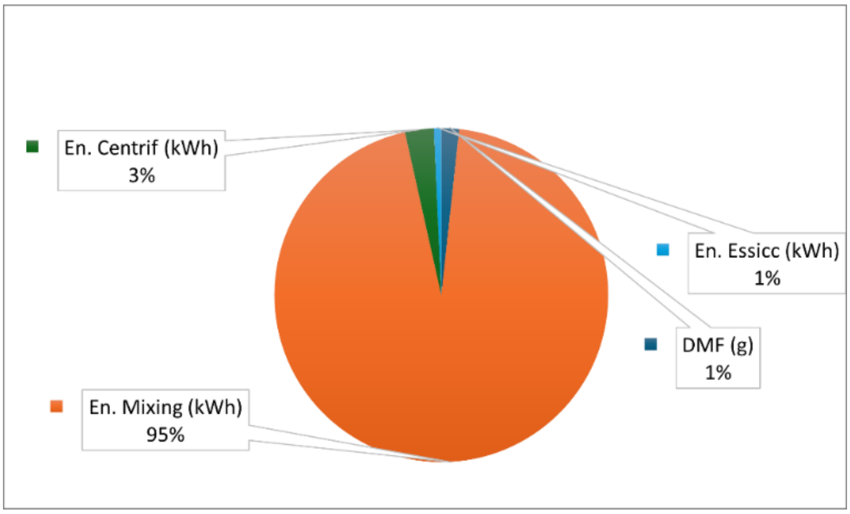

Figure S6.1 Contribution analysis expressed in cumulative single score through the ReCiPe EndPoint H approach for Z-DMF.

S7. Uncertainity analysis

Table S7.1 Pedigree Matrix value for both Z-DMF and Z-GlyC

Item	Reliability	Completeness	Temporal correlation	Geographical correlation	Further tecnological correlation
Z-DMF					
2-MIm	2	2	4	1	3
Zinc nitrate	2	2	4	1	4
DMF	2	2	4	1	1
Electricity - low voltage	2	2	4	1	4
Washing with MeOH	2	2	4	1	2
Z-GlyC					
2-Mim	2	2	4	1	3
Zn acetate	2	1	1	1	4
NaOH	2	1	1	1	1
glicerolo_rapeoil	1	1	1	1	1
glicerolo_wastecookingoil	1	2	1	2	1
DMC	1	1	1	1	1
Na2CO3	1	1	1	1	1
GlyC	2	4	1	1	4
Electricity - low voltage-GlyC synthesis	2	2	1	1	4
Incineration	2	2	1	1	2
Washing with MeOH	1	1	1	1	1
Electricity - GlyC distillation	2	2	1	1	4
Ultrasonic - electricity	2	2	1	1	1
Elecricity for ZIF8 synthesis	2	2	1	1	4

Table S7.2 SD^2 values for both Z-DMF and Z-GlyC

Item	SD^2 (σ^2)
	Z-DMF
2-MIm	1.2
Zinc nitrate	1.3
DMF	1.2
Electricity - low voltage	1.3
Washing with MeOH	1.2
	Z-GlyC
2-Mim	1.2
Zn acetate	1.2
NaOH	1.1
glicerolo_rapeoil	1.1
glicerolo_wastecookingoil	1.1
DMC	1.1
Na2CO3	1.1
GlyC	1.3
Electricity - low voltage-GlyC synthesis	1.2
Incineration	1.1
Washing with MeOH	1.1
Electricity - GlyC distillation	1.2
Ultrasonic - electricity	1.1
Elecricity for ZIF8 synthesis	1.2

Monte Carlo analysis

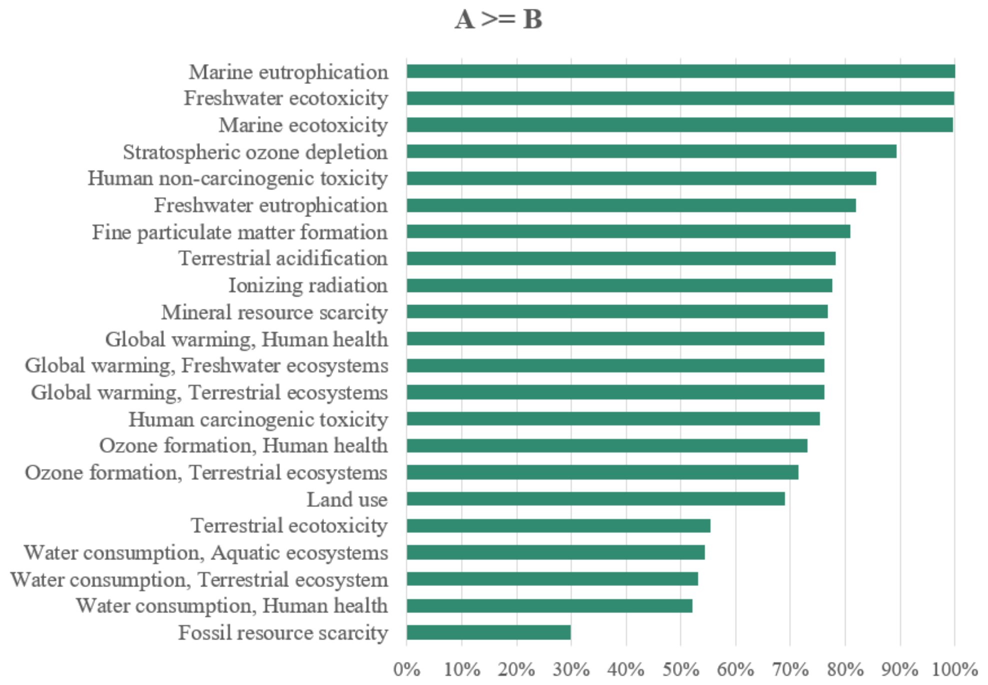

Figure S7.3 Uncertainty analysis of 1 g 'Z-DMF' (A) minus 1 g 'Z-GlyC-best' (B). Method: ReCiPe 2016 Endpoint (H) V1.05 / World (2010) H/A , confidence interval: 95 %

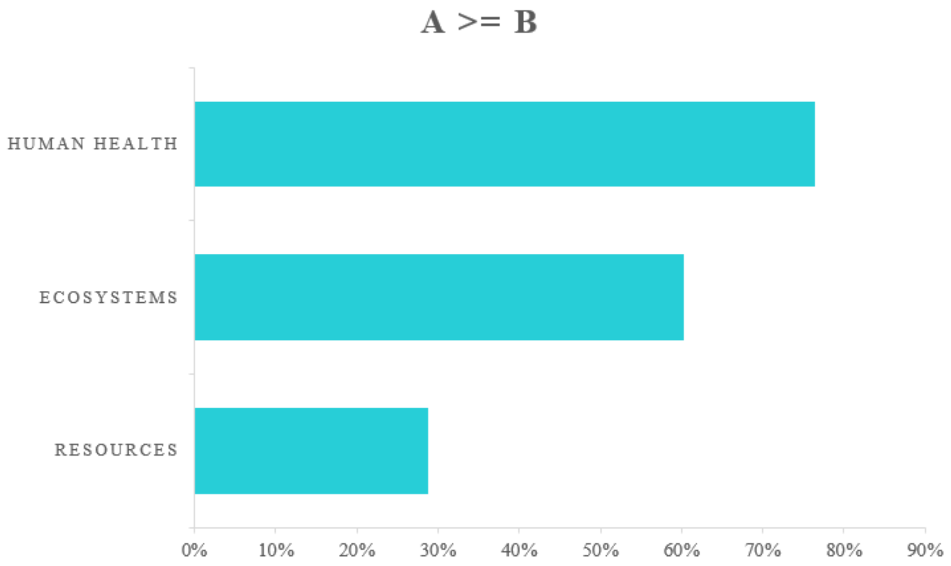

Figure S7.4 Uncertainty analysis of 1 g 'Z-DMF' (A) minus 1 g 'Z-GlyC-best' (B). Method: ReCiPe 2016 Endpoint (H) V1.05 / World (2010) H/A , confidence interval: 95 %

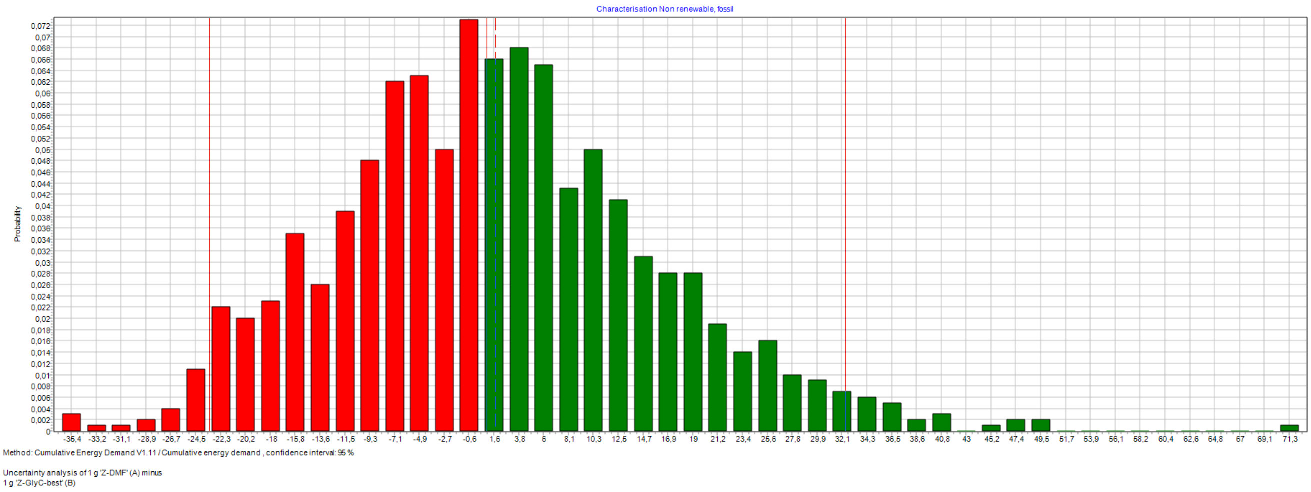

54.3% probability for A>=B

Figure S7.5 Uncertainty analysis of Z-DMF > Z-GlyC-best.

Method: Cumulative energy demand V1.1, confidence interval 95%.
